# Shade, light, and stream temperature responses to riparian thinning in second-growth redwood forests of northern California

**DOI:** 10.1371/journal.pone.0246822

**Published:** 2021-02-16

**Authors:** David A. Roon, Jason B. Dunham, Jeremiah D. Groom

**Affiliations:** 1 Department of Fisheries and Wildlife, Oregon State University, Corvallis, Oregon, United States of America; 2 U.S. Geological Survey, Forest and Rangeland Ecosystem Science Center, Corvallis, Oregon, United States of America; 3 Groom Analytics, LLC, Corvallis, Oregon, United States of America; Universite du Quebec a Chicoutimi, CANADA

## Abstract

Resource managers in the Pacific Northwest (USA) actively thin second-growth forests to accelerate the development of late-successional conditions and seek to expand these restoration thinning treatments into riparian zones. Riparian forest thinning, however, may impact stream temperatures–a key water quality parameter often regulated to protect stream habitat and aquatic organisms. To better understand the effects of riparian thinning on shade, light, and stream temperature, we employed a manipulative field experiment following a replicated Before-After-Control-Impact (BACI) design in three watersheds in the redwood forests of northern California, USA. Thinning treatments were intended to reduce canopy closure or basal area within the riparian zone by up to 50% on both sides of the stream channel along a 100–200 m stream reach. We found that responses to thinning ranged widely depending on the intensity of thinning treatments. In the watersheds with more intensive treatments, thinning reduced shade, increased light, and altered stream thermal regimes in thinned and downstream reaches. Thinning shifted thermal regimes by increasing maximum temperatures, thermal variability, and the frequency and duration of elevated temperatures. These thermal responses occurred primarily during summer but also extended into spring and fall. Longitudinal profiles indicated that increases in temperature associated with thinning frequently persisted downstream, but downstream effects depended on the magnitude of upstream temperature increases. Model selection analyses indicated that local changes in shade as well as upstream thermal conditions and proximity to upstream treatments explained variation in stream temperature responses to thinning. In contrast, in the study watershed with less intensive thinning, smaller changes in shade and light resulted in minimal stream temperature responses. Collectively, our data shed new light on the stream thermal responses to riparian thinning. These results provide relevant information for managers considering thinning as a viable restoration strategy for second-growth riparian forests.

## Introduction

Riparian forests provide numerous ecosystem functions for their associated stream and river systems [1, 2]. Due to these linkages, changes in riparian forest conditions can directly affect adjacent stream ecosystems [3].

After decades of unregulated timber harvest practices that removed riparian forests from forested landscapes in the Pacific Northwest (USA), riparian buffers now largely protect riparian forests [4, 5]. As a result, buffer protections that limit forest harvest in riparian zones have successfully restored some key ecological functions [2, 4, 5]. However, riparian management policies continue to vary by state, species presence (fish-bearing or not), and landownership, and their implementation tends to reflect the goal of administrative simplicity (i.e., uniform buffer widths) rather than taking into account local ecological context, all of which may limit their effectiveness [6–8].

Riparian forests protected by buffers often reflect the legacy of past land-use [9]. Dense, even-aged stands of early-seral species tend to dominate regenerating forests within riparian buffers and therefore these forests differ in structure and composition from the old-growth forests that preceded them [10, 11]. To address this, resource managers have expressed interest in more active management within riparian zones [12, 13]. For example, federal land managers in the Pacific Northwest are exploring the application of silvicultural methods such as selective logging and variable density thinning as restoration strategies to accelerate the development of late-successional forest structure and composition [14]. Moreover, managers are interested in understanding whether thinning accelerates the recovery of large conifers in riparian forests to provide an eventual source of large woody debris, promote riparian zone heterogeneity, and enhance aquatic and riparian biodiversity and productivity [15–17]. As a result, there is a growing interest in thinning as a restoration strategy to address multiple objectives for second-growth riparian forests impacted by previous land-use.

Forest restoration is a key concern in the coast redwood forests (*Sequoia sempervirens*) of northern California. Only 3–5% of old-growth redwood forests remain in this region and have been largely replaced by dense second-growth stands often dominated by commercially-planted species such as Douglas-fir (*Pseudotsuga menziesii*) or early-successional species such as red alder (*Alnus rubra*) [18, 19]. Resource managers actively thin these forests in an attempt to promote the recovery of old-growth redwood forests and increase heterogeneity of dense-second growth stands [20, 21]. To date, restoration thinning treatments have targeted upland forests, but as the composition and structure of second-growth riparian forests appear similarly affected by previous harvest [18, 19], there is interest in expanding thinning activities into riparian zones. However, given that changes in riparian forest conditions can affect adjacent stream ecosystems [3], it is important to understand the effects of riparian thinning on streams.

Changes in riparian forests can influence stream conditions in many ways, but the most immediate responses to canopy removal include changes in shading, solar radiation, and stream temperature [4]. Stream thermal conditions are primary drivers of ecological processes in aquatic ecosystems [22, 23]. Large-scale reductions in riparian shade associated with historical timber harvest practices such as clearcutting frequently led to warming stream temperatures that exceeded the thermal tolerance of Pacific Northwest cold-water adapted stream fishes and amphibians [4, 24, 25]. As a result, state and federal policies now limit the magnitude of change in stream temperature caused by land-use activity like timber harvest [4]. Recent research suggests that contemporary forest management practices that include riparian buffers often effectively prevent temperature increases, although substantial variability and context dependence have been documented [4, 26–29].

Recent studies of stream temperature responses to contemporary forestry provide critical insights, yet many knowledge gaps remain surrounding the effects of forest thinning in riparian zones. First, in comparison to the information available on the effects of historical forestry practices [4], we know little about the effects of more subtle changes in shade and light associated with thinning second-growth riparian forests. Most studies to date have evaluated the effects of clearcutting with no buffer or riparian harvests outside of an untouched buffer [4, 14]. In contrast, few studies have quantified the effects of thinning within riparian buffers near streams. Second, regulatory requirements focus on single descriptors of stream temperature (e.g., summer maxima), which may inadequately describe thermal influences on ecological processes [30]. Recent studies that characterize stream temperature as a *thermal regime* including the magnitude, variability, duration, frequency, and timing have been effective in developing a more comprehensive understanding of stream thermal conditions [31–33]. However, few studies have applied this approach to understand how stream thermal regimes respond to disturbance such as forest management [34].

In this study we evaluated the effects of riparian thinning on shade, light, and stream temperature in a manipulative field experiment following a Before-After-Control-Impact (BACI) study design [35] in three watersheds located in the second-growth redwood forests of northern California. The objectives of this study were to evaluate: 1) the effects of experimental riparian thinning treatments on shade and light conditions; 2) how changes in shade and light associated with thinning affected stream temperatures at a reach-scale both locally and downstream; 3) how thermal responses varied seasonally; and 4) how these thermal responses were expressed across the broader thermal regime to gain a more complete understanding of thinning on stream temperatures in these watersheds.

BACI designs are effective in detecting changes and have been applied in many similar experiments (e.g., [26, 29]). However, further investigation is needed to disentangle the environmental factors responsible for driving observed responses. Therefore, we also employed additional analyses to evaluate the underlying relationships hypothesized to influence stream heat budgets [4, 23]. For this series of analyses, we used correlation and model selection approaches [36] to evaluate a suite of models to determine which best explained the variability in different descriptors of stream thermal regimes and their responses to thinning.

## Methods

### Study systems

**Fig 1:**
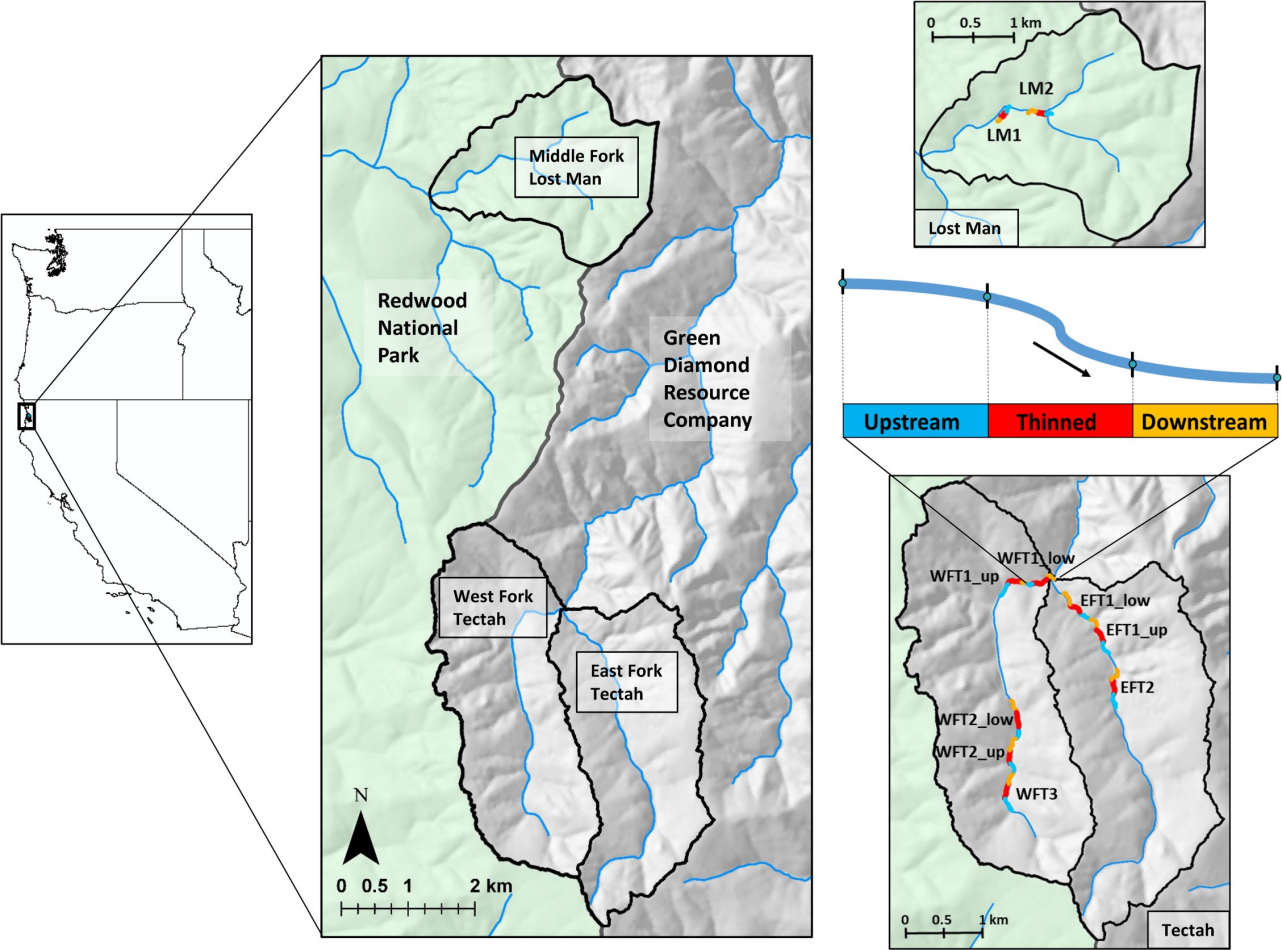
Study watersheds map. Map of study watersheds in northern California second-growth redwood forests. Data were collected in upstream reference, thinned, and downstream reaches, which were replicated at 10 total sites distributed across these three watersheds. Temperature sensors were deployed at the top and bottom of each reach indicated by blue points in illustration of study reaches. See Table 1 for characteristics of study sites. Map by David A. Roon in ArcGIS Pro (ESRI, Redlands, CA USA) using data collected by the authors and publicly-available GIS shapefiles from the California State Geoportal: https://gis.data.ca.gov/ [41, 42].

We conducted this study in three watersheds located in the coast redwood forests of northern California (Fig 1). The West Fork Tectah and East Fork Tectah watersheds were located on private timber land owned by Green Diamond Resource Company and flow into the lower Klamath River. The Lost Man watershed was located in Redwood National Park and flowed into Prairie Creek, a major tributary to Redwood Creek. All three watersheds were drained by low-order streams (watershed areas 5.8–8.4 km^2^) located within 15 km of the Pacific Ocean and experienced a temperate, maritime climate heavily influenced by coastal fog [37]. Riparian forests bordering these streams were primarily composed of dense second-growth forests regenerating from timber harvest 40–60 years ago. Riparian canopies consisted of a mix of red alder, coast redwood, Douglas-fir, western hemlock (*Tsuga heterophylla*), tanoak (*Notholithocarpus densiflorus*), and western red cedar (*Thuja plicata*). Flow regimes in these systems are highly seasonal, driven by frequent coastal rainstorms that result in high flows during winter months followed by descending summer low flows supplemented by coastal fog, upwelling groundwater, and hyporheic flow [38]. Resident populations of coastal cutthroat trout (*Oncorhynchus clarkii clarkii*), coastal giant salamander (*Dicamptodon tenebrosus*), and coastal tailed frog (*Ascaphus truei*) occupy these watersheds and are sensitive to increases in stream temperature [39, 40].

### Experimental design

We collected data for this study as part of a manipulative field experiment following a replicated BACI design. We collected pre-treatment data in 2016, experimental thinning treatments occurred in 2017, and we collected post-treatment data in 2017 and 2018. We collected post-treatment riparian shade and light data in 2017 and 2018, but we limited the analysis of post-treatment stream temperature data to 2018 due to the staggered timing of the thinning treatments. Rather than establishing a control watershed as in many stream temperature studies (e.g., [27, 29]), to account for inherent spatial heterogeneity within and between watersheds all experimental thinning reaches (130–225 m in length) immediately bordered an upstream reference reach, similar to the design described in Groom et al. [26]. To determine the longitudinal persistence of local thermal responses associated with thinning we also monitored conditions in downstream reaches ~150–200 m in length. We replicated this three-reach design at 10 total sites distributed across these three watersheds (Table 1). We did not randomly select the study sites, so the results have limited inference to the Tectah and Lost Man watersheds, but may also apply to similar locations in the northern California redwood forests. Our sites sometimes occurred sequentially along the three stream channels and therefore not all sites were statistically independent. However, we included variables in our analysis to account for spatial non-independence.

**Table 1:** Study site characteristics.

Watershed	Site ID	Distance Upstream (m)	Reach Length (m)	Bankfull Width (m)	Aspect (°)	Gradient (%)	Elevation (m)
West Fork Tectah	WFT1_lower	140	225	6.6	75	1.4	351.3
	WFT1_upper	535	175	6.0	90	2.2	359.2
	WFT2_lower	2750	205	4.7	350	2.9	409.0
	WFT2_upper	3320	195	3.7	15	3.2	429.2
	WFT3	3840	220	3.2	25	6.4	456.5
East Fork Tectah	EFT1_lower	450	195	5.3	310	3.9	363.5
	EFT1_upper	990	170	6.1	325	5.3	385.5
	EFT2	1850	225	4.6	345	3.8	421.3
Lost Man	LM1	1450	130	4.5	220	2.8	357.8
	LM2	2300	140	4.1	275	3.1	368.8

Physical site characteristics of experimental thinning reaches (n = 10) distributed across three study watersheds–West Fork Tectah, East Fork Tectah, and Lost Man in northern California second-growth redwood forests. Distance Upstream indicates watershed position as distance upstream (in meters) from the confluence.

The landowners included in this study followed distinct riparian thinning treatment prescriptions tailored to their management objectives.

In the Tectah watersheds on Green Diamond Resource Company property, thinning prescriptions intended to reduce overstory canopy closure within the riparian zone to 50% on both sides of the channel along a ~200 m stream reach. Thinning treatments targeted red alder and some conifers depending on the composition and density of the stand, but left all large conifers that would contribute as an eventual source of large woody debris. Thinning treatments occurred next to upslope timber harvest units and trees were removed from the riparian zone via cable yarding. Upstream reference and downstream reaches were bordered by either intact forest on both sides of the stream channel or, when within harvest units, by intact forest on one side and a riparian buffer following Green Diamond’s standard buffer prescription on the harvest side of the stream. The one-sided buffer prescription consisted of 45 m wide buffer with a 22.5 m inner zone of 85% canopy retention and a 22.5 m outer zone of 70% canopy retention as prescribed by Green Diamond’s Aquatic Habitat Conservation Plan with the National Marine Fisheries Service and United States Fish and Wildlife Service [43]. Although upstream and downstream reaches occurred both inside and outside harvest units, we documented no difference in shade, light, and stream temperature conditions in an analysis of reference reach types, allowing us to group them together (S1 File).

In the Lost Man watershed in Redwood National Park (RNP) riparian thinning treatments coincided with a larger upland forest restoration thinning effort in the Middle Fork of the Lost Man Creek watershed [44]. Riparian thinning treatments sought to remove up to 40% of the basal area within the riparian zone on slopes less than 20% on both sides of the channel along a ~100–150 m reach. Riparian thinning treatments primarily targeted Douglas-fir and red alder to achieve RNP’s objective of promoting the recovery of late-successional coast redwood forests [21]. While thinning treatments removed trees from upland forests, trees within the riparian zone were felled following a lop-and-scatter protocol which left trees in the riparian zone but out of the stream channel.

### Riparian shade

We measured riparian shade over the stream channel using hemispherical photography following the methods described in Ringold et al. [45]. We took hemispherical photographs with a Canon EOS 70D digital camera (Canon Inc., Tokyo, Japan) equipped with a circular fisheye lens attached to a leveled tripod and oriented to north. To characterize shade within upstream reference, thinned, and downstream reaches during leaf-out conditions, we took photographs mid-summer each year 1 m above the stream channel every 10 m at mid-bankfull width (n = 10–22 photographs/reach). We took photographs early in the morning and under a range of exposures to ensure that direct sunlight would not interfere with shade characterization. We analyzed photographs in HemiView Canopy Analysis Software version 2.1 (Delta-T Devices 1998), which classifies light and dark pixels to quantify shade. In HemiView, we selected two output metrics to characterize riparian shade: 1) canopy closure–which considers the total amount of shade in the entire photograph using the formula: Canopy Closure (%) = (1-VisSky)*100, where VisSky represents the total number of “open” pixels visible to the sky; and 2) effective shade–which considers the amount of shade that covers the solar pathway over that location using the formula: Effective Shade (%) = (1-GSF)*100, where GSF (Global Site Factor) is the number of “open” pixels within the path of the sun.

### Light

We measured solar radiation reaching the stream channel using silicon pyranometers (Onset Solar Radiation Smart Sensor, Onset Computer Corporation, Bourne, MA USA), which detect a broad spectrum of light (300–1100 nm). To measure the amount of solar radiation available above the forest canopy, we deployed a weather station equipped with a pyranometer attached to an Onset Micro Station (Onset Computer Corporation, Bourne, MA USA) on a ridge nearby each watershed. To measure the amount of solar radiation that filters through the canopy to the stream in upstream reference, thinned, and downstream reaches mid-summer, we deployed four pyranometers within each reach at 25 m intervals 1 m above the stream attached to a central Onset Micro Station for 24 hours during the same time of year that we took hemispherical photographs. Pyranometers recorded solar radiation hourly during this 24-hour window. We expressed the amount of light that filtered through the canopy to the stream as a percentage of the amount of above-canopy light available.

### Stream temperature

We measured stream temperatures using digital temperature sensors (a combination of Onset Hobo Water Temperature Pro v2 and TidbiT Water Temperature Data Loggers, Onset Computer Corporation, Bourne, MA USA). Before deployment we checked that all sensors were properly calibrated following Heck et al. [46]. We protected sensors from solar radiation using solar shields constructed from 5 cm diameter polyvinyl chloride (PVC) pipe ~13cm in length. We anchored sensors to the streambed using Duckbill Earth Anchors (MacLean Civil Products, Fort Mill, SC, USA) modified with 5mm diameter vinyl-coated galvanized steel cables in gravel and cobble dominant habitats or with waterproof epoxy (Pettit Splash Zone Marine Epoxy, Pettit Paint, Rockaway, NJ, USA) in habitats where bedrock or large boulders predominated. We deployed temperature sensors at the upstream and downstream extent of upstream reference, thinned, and downstream reaches. We deployed sensors in the Tectah watersheds in fall of 2015 and in the Lost Man watershed in spring of 2016. We then monitored stream temperature hourly through the end of the 2018 water year.

To develop a more comprehensive understanding of how thinning influenced stream thermal regimes, we characterized stream temperature responses using a suite of descriptors described in Arismendi et al. [32] and Benjamin et al. [47]. To determine the extent to which temperature increased, we used the following descriptors of magnitude: average daily maximum, the maximum weekly average of the maximum (MWMT), average daily mean, the maximum weekly average of the mean (MWAT), cumulative seasonal degree days, and average daily minimum. To determine how the distribution and spread of stream temperature changed, we used the following descriptors of variability: average daily range, maximum daily range, average variance, and maximum variance. To determine the temporal frequency and duration of these temperature changes above common regulatory cold-water thresholds [47, 48], we used the following frequency and duration descriptors: number of days where daily temperature > 16°C, the number of consecutive days > 16°C, the number of days > 20°C, and the number of consecutive days > 20°C. To determine the timing of temperature responses, we noted the seasonal occurrence during the water year (Fall, Winter, Spring, Summer). In order to calculate these descriptors, hourly temperature data were summarized as daily values. Daily values were then summarized seasonally for all responses described above. We defined seasonal windows by the start of the water year (Oct 1 –Sep 30) and by the inherent seasonal patterns of thermal conditions in these streams: Fall (Oct 1 –Dec 31), Winter (Jan 1 –Mar 31), Spring (Apr 1 –Jun 30), and Summer (Jul 1 –Sep 30).

## Data analyses

Due to the differences in thinning treatment prescriptions we evaluated Tectah and Lost Man separately for each analysis. Because temperature sensors were deployed in the spring of 2016 in the Lost Man watershed, we limited before-after analyses for Lost Man to spring and summer unless specified. All analyses were conducted in R [49].

### BACI analysis

We conducted a classic BACI analysis [35] to evaluate the effects of thinning on riparian shade, light, and stream temperature using linear mixed-effects models [50] in the nlme package in R [51]. To do this, we used mean estimates of response variables for upstream reference, thinned, and downstream reaches from our pre-treatment and post-treatment years using the fixed-effects model:

BACImodel:ResponseVariable∼Reach+Year+Reach*Year+ε

This BACI model tests whether the response variable is explained by Reach (upstream reference vs. thinned vs. downstream), Year (pre-treatment vs post-treatment), and the interaction of Reach*Year (BACI effect). Under this design, a significant BACI effect of Reach*Year effect indicates an effect of thinning (α = 0.05). To account for the variation between sites, we included a random intercept by Site and a weights argument to relax the assumption of constant variance among Reaches and Years [50]. Remaining unexplained error is represented by ε. We then estimated BACI differences for thinned and downstream reaches following the formulas:

$$BACI\;difference\;for\;thinned\;reaches:\;\left(Thinned_{Post}-Thinned_{Pre}\right)-\left(Upstream_{Post}-Upstream_{Pre}\right)$$

$$BACI\;difference\;for\;downstream\;reaches:\;\left(Downstream_{Post}-Downstream_{Pre}\right)-\left(Upstream_{Post}-Upstream_{Pre}\right)$$

We estimated the BACI differences and 95% confidencintervals. If 95% confidence intervals did not overlap 0, we considered the effect to be statistically significant. We checked the residuals for all BACI models to make sure we met assumptions of constant variance and normality [50].

### Longitudinal profiles

We plotted reach-scale longitudinal profiles of MWMT following the methods described in Arismendi and Groom [52] to visualize how local temperature increases associated with thinning propagated downstream. To do this, we set the pre-post difference in temperature (MWMT) for the sensor at the upstream end of the upstream reference reach to 0 to standardize comparisons of temperature responses between sites for upstream, thinned, and downstream reaches. We then repeated longitudinal profiles of each site for each season to visualize seasonal variability of temperature responses in thinned and downstream reaches.

### Multivariate analyses of thermal regimes

We applied multivariate analyses to evaluate how the structure of stream thermal regimes responded to thinning. We used non-metric multidimensional scaling (NMS) ordination to visualize how the structure of the entire thermal regime (including multiple descriptors for magnitude, variability, duration, and frequency) varied. To do this, we created a matrix for selected descriptors (n = 16) of our stream thermal regimes for all reaches and sites for our pre-treatment and post-treatment years for each season (n = 220 total combinations). We then ran NMS ordinations using a Euclidean distance measure that followed an iterative optimization procedure (n = 999 times) [32]. We checked the final solution against goodness of fit tests and for overall stress and displayed the results in two dimensions. We added ellipses indicating the 95% confidence intervals for each reach (upstream reference vs. thinned vs. downstream) to indicate how the structure of thermal regimes varied between reaches during each season. We then applied permutational multivariate analysis of variance (PerMANOVA) to test whether the structure of stream thermal regimes differed due to thinning indicated by a significant BACI effect of Reach*Year (α = 0.05). All multivariate analyses were conducted in the vegan package in R [53].

### Environmental factors

We further explored the environmental factors driving the variation in thermal responses associated with thinning by evaluating the role of shade, light, and other environmental covariates. First, we related BACI responses in summer MWMT to the responses in shade and light associated with thinning for all sites. Second, we evaluated the correlations between summer MWMT and environmental covariates frequently considered in stream temperature studies [4, 23, 26, 28]. Environmental covariates included: shade, light, upstream temperatures, air temperature, proximity to upstream treatments, as well as physical site characteristics such as distance upstream, reach length, bankfull width, gradient, aspect, and elevation. See S2 Table for more detailed descriptions of the environmental covariates we considered. Correlations were assembled in a correlation matrix using the corrplot package in R [54].

### Model selection

To better understand the factors driving the variability in different descriptors of stream thermal regimes and their responses to thinning, we applied a model selection approach following the methods provided by Burnham and Anderson [36] and Zuur et al. [50]. Model selection focused on summer conditions when most covariates were available. We used a correlation matrix to guide the fixed effects we included in our candidate models and avoided covariates that were highly correlated (*r* > 0.6) or represented similar ecological processes (S1 Fig). We also checked variance inflation factor scores of the variables within candidate models to ensure that multicollinearity did not occur between variables. Starting with a “beyond optimal” fully overparameterized model we selected a random effects structure. Random effects were fit using Restricted Maximum Likelihood. We then ranked candidate sets of *a priori* models exploring different fixed effects using AIC_c_ values in the AICcmodavg package in R [55]. Fixed effects models were fit using Maximum Likelihood. The best supported model determined by the lowest AIC_c_ value was then refit with Restricted Maximum Likelihood to obtain unbiased parameter estimates. We checked the residuals for all best supported models to make sure we met assumptions of constant variance and normality for fixed and random effects [50]. See S2 Table for the full list of models considered in candidate model sets. Best supported models for each response variable are listed in S3 Table.

## Results

### BACI analysis—riparian shade

**Fig 2:**
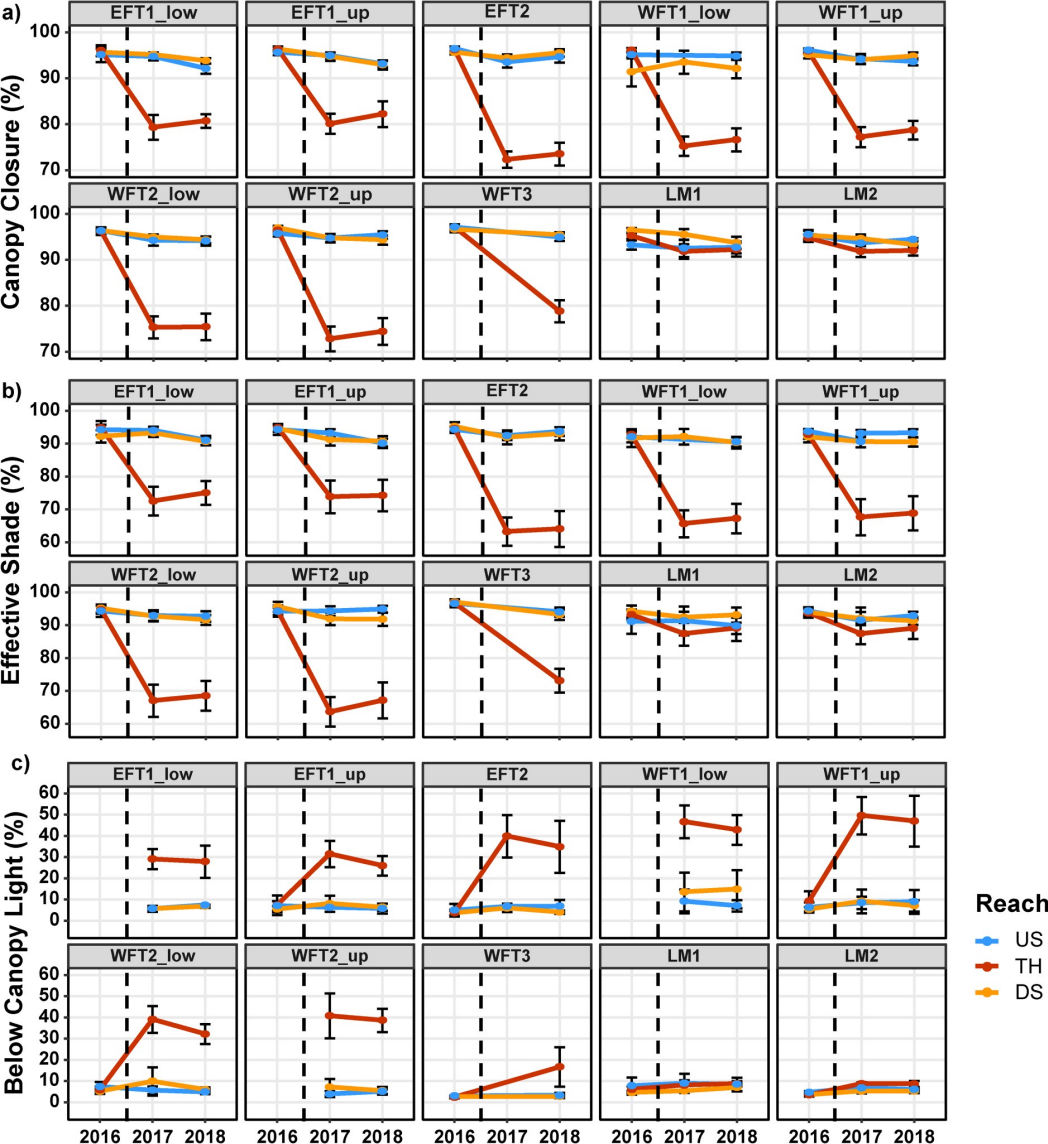
Riparian shade and light responses to riparian thinning. Riparian shade and light responses to riparian thinning in northern California second-growth redwood forests. Riparian shade summarized as a) canopy closure (%), b) effective shade (%), and solar radiation summarized as c) below-canopy light (%) for Tectah and Lost Man sites. Data collected in upstream reference (US), thinned (TH), and downstream (DS) reaches during pre-treatment (2016) and post-treatment years (2017 and 2018) for each site. Points indicate mean estimates with error bars indicating 95% confidence intervals. Vertical hashed line indicates the timing of experimental thinning treatments.

Pre-treatment estimates of riparian shade in 2016 indicated uniformly high levels of canopy closure and effective shade across all three reach types (upstream reference, thinned, and downstream) and did not differ among reaches in the Tectah or Lost Man watersheds (Fig 2). Post-treatment estimates in 2017 and 2018 indicated that riparian thinning treatments decreased riparian shade, but the extent of reductions varied between watersheds (Fig 2, S1 Table). In the Tectah watersheds, BACI models indicated that riparian shade decreased significantly in thinned reaches as canopy closure by a mean of 18.7% (95% confidence intervals: -21.0, -16.3) in 2017 and 16.9% (-19.2, -14.6) in 2018 and as effective shade by a mean of 25.4% (-28.6, -22.3) in 2017 and 23.0% (-25.8, -20.1) in 2018 (Fig 2, S1 Table). In the Lost Man watershed, effective shade decreased in thinned reaches by a mean of 4.8% (95% confidence intervals: -8.0, -0.5) in 2017 and 4.1% (-8.3, -0.3) in 2018, but BACI models determined that reductions in canopy closure (-2.1% in 2017 and -1.9% in 2018) were not significant in either year (Fig 2, S1 Table).

### BACI analysis—light

Pre-treatment estimates of below-canopy light in 2016 documented that only a small portion of the overall solar radiation available (~6%) filtered through the canopy to the stream channel and did not differ among reaches in the Tectah or Lost Man watersheds (Fig 2). Post-treatment estimates in 2017 and 2018 indicated that riparian thinning increased below-canopy light, but the intensity of responses varied between watersheds (Fig 2, S1 Table). In the Tectah watersheds, BACI models indicated that below-canopy light increased significantly in thinned reaches by a mean of 33.0% (95% confidence intervals: 27.3, 38.5) in 2017 and 27.1% (20.4, 33.8) in 2018 (Fig 2, S1 Table). In the Lost Man watershed, we observed below-canopy light increase slightly in thinned reaches by a mean of 2.9% (-0.7, 6.5) in 2017 and 2.5% (-1.6, 5.6) in 2018, but BACI models determined that these increases were not statistically significant (Fig 2, S1 Table).

### BACI analysis—stream temperature

**Fig 3:**
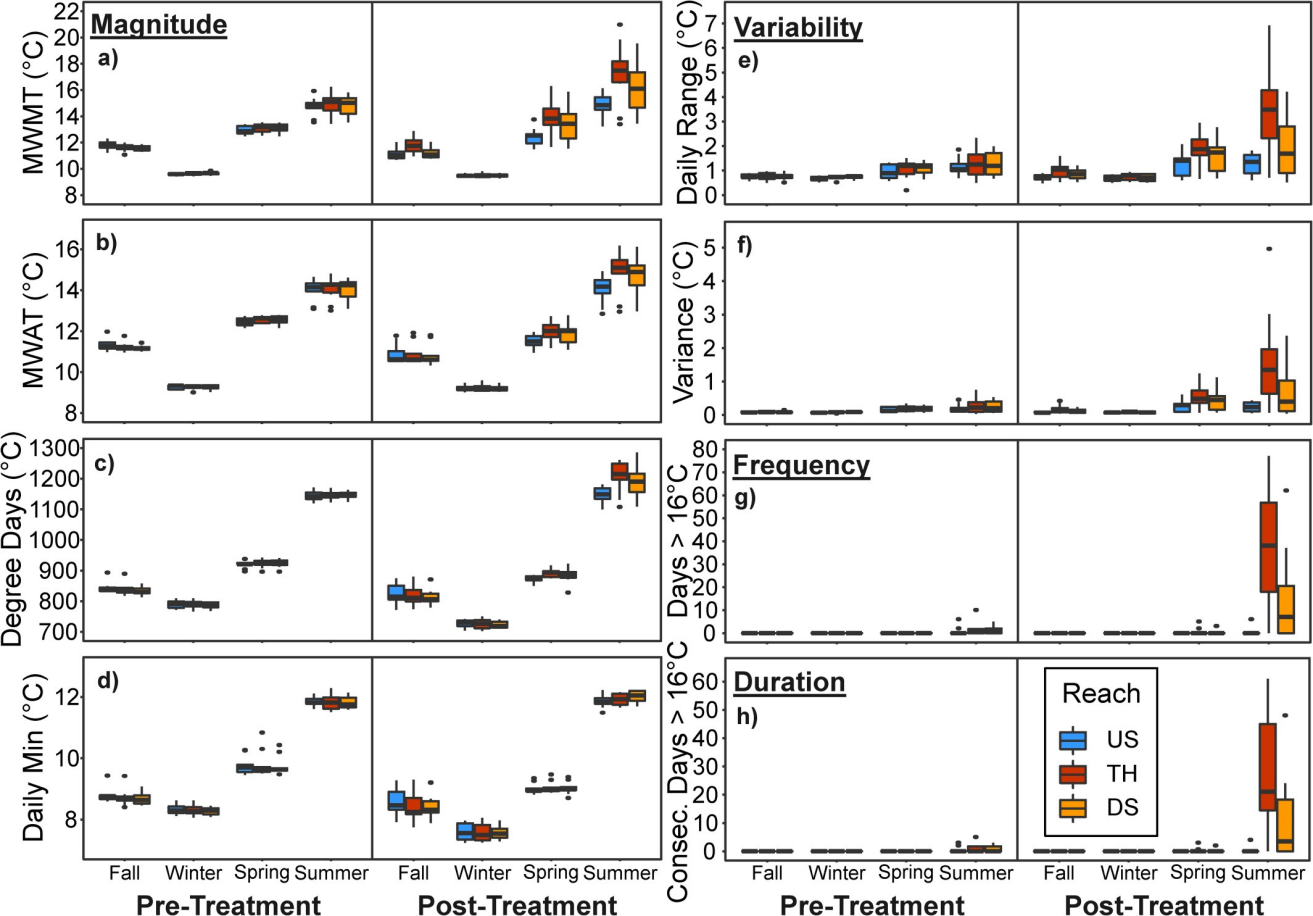
Stream thermal regime responses to riparian thinning. Seasonal patterns in stream temperature in upstream reference (US), thinned (TH), and downstream (DS) reaches during pre-treatment (2016) and post-treatment (2018) water years in northern California second-growth redwood forests. Boxplots show the distribution of responses across all sites (n = 10) for selected stream thermal regime descriptors including: magnitude (a-d), variability (e-f), frequency (g), and duration (h). Stream temperature responses were summarized for each seasonal window: Fall (October-December), Winter (January-March), Spring (April-June), and Summer (July–September).

Stream temperatures varied seasonally for each descriptor of the thermal regime we considered (magnitude, variability, frequency, and duration) during the pre-treatment water year, but did not differ between reaches for any descriptor (Fig 3).

Riparian thinning increased the magnitude of stream thermal regimes in thinned and downstream reaches, but responses varied seasonally and between watersheds (Fig 3, Tables 2 and 3). In the Tectah watersheds, BACI models indicated that responses in magnitude were most pronounced as changes in maximum temperatures, which increased MWMT in thinned reaches during spring by a mean of 1.7°C (95% confidence intervals: 0.9, 2.5), summer by a mean of 2.8°C (1.8, 3.8), and fall by a mean of 1.0°C (0.5, 1.5) and increased in downstream reaches during spring by a mean of 1.0°C (0.0, 2.0) and summer by a mean of 1.4°C (0.3, 2.6) (Tables 2 and 3, S1 Table). BACI models indicated that thinning increased mean temperatures as MWAT and cumulative seasonal degree days in thinned reaches during spring (MWAT: 0.5°C; degree days 18.6°C) and summer (MWAT: 0.9°C; degree days: 77.7°C) and in downstream reaches during summer (MWAT: 0.6°C; degree days: 48.1°C) (Tables 2 and 3, S1 Table). We observed no change in minimum temperatures (Fig 3, Tables 2 and 3). In the Lost Man watershed, BACI models indicated no effect of thinning on stream temperatures in thinned or downstream reaches for any descriptor of magnitude during any season (Tables 2 and 3, S1 Table).

**Table 2:** Stream thermal regime responses in thinned reaches.

Temperature Response (Δ°C)	Watershed	Fall	Winter	Spring	Summer
*Magnitude*			** **	** **	** **
Daily Maximum	Tectah:	1.4 (1.0, 1.8)	0.1 (-0.1, 0.2)	1.7 (1.1, 2.3)	2.9 (2.1, 3.6)
	Lost Man:	0.1 (0.1, 0.2)	0.1 (0.0, 0.1)	0.4 (0.1, 0.6)	0.3 (0.1, 0.5)
MWMT	Tectah:	1.0 (0.6, 1.4)	-0.1 (-0.1, 0.1)	1.6 (1.0, 2.2)	2.7 (2.0, 3.4)
	Lost Man:	0.1 (-0.1, 0.1)	0.1 (0.0, 0.1)	0.1 (-0.2, 0.5)	0.2 (-0.1, 0.4)
Daily Mean	Tectah:	0.0 (-0.1, 0.1)	0.0 (-0.1, 0.1)	0.2 (0.1, 0.3)	0.8 (0.6, 1.0)
	Lost Man:	0.1 (0.0, 0.1)	0.1 (0.0, 0.1)	-0.2 (-0.6, 0.1)	0.2 (0.1, 0.2)
MWAT	Tectah:	0.1 (0.0, 0.2)	0.0 (-0.1, 0.1)	0.5 (0.3, 0.6)	0.9 (0.6, 1.1)
	Lost Man:	0.1 (0.0, 0.1)	0.1 (0.0, 0.1)	0.0 (-0.2, 0.1)	0.2 (0.1, 0.3)
Degree Days	Tectah:	3.3 (-3.0, 9.6)	0.7 (-2.2, 3.5)	17.1 (8.7, 23.4)	73.4 (56.5, 90.3)
	Lost Man:	5.6 (-0.7, 11.9)	5.8 (2.7, 8.9)	6.8 (6.1, 7.5)	13.8 (9.5, 18.1)
Daily Minimum	Tectah:	-0.1 (-0.2, 0.0)	0.0 (-0.1, 0.0)	0.0 (0.0, 0.1)	0.1 (-0.1, 0.3)
	Lost Man:	0.0 (-0.1, 0.1)	0.1 (0.0, 0.1)	-0.1 (-0.2, 0.1)	0.1 (0.0, 0.1)
*Variability*					
Average Daily Range	Tectah:	0.4 (0.2, 0.5)	0.0 (-0.1, 0.1)	0.5 (0.3, 0.8)	2.4 (1.8, 3.0)
	Lost Man:	0.0 (0.0, 0.1)	0.0 (0.0, 0.1)	0.2 (0.0, 0.4)	0.1 (0.0, 0.1)
Maximum Daily Range	Tectah:	1.2 (0.7, 1.8)	-0.1 (-0.4, 0.2)	1.5 (0.7, 2.2)	2.9 (2.0, 3.8)
	Lost Man:	0.0 (0.0, 0.0)	0.1 (0.0, 0.3)	0.2 (0.1, 0.2)	0.1 (0.1, 0.2)
Average Variance	Tectah:	0.1 0.1 (0.1, 0.2)	0.0 (-0.1, 0.1)	0.3 (0.1, 0.5)	1.5 (0.9, 2.3)
	Lost Man:	0.0 (0.0, 0.0)	0.0 (0.0, 0.0)	0.0 (0.0, 0.0)	0.0 (0.0, 0.1)
Maximum Variance	Tectah:	0.7 (0.3, 1.2)	-0.1 (-0.1, 0.1)	1.2 (0.5, 2.0)	2.4 (1.4, 3.7)
	Lost Man:	0.0 (0.0, 0.1)	0.0 (0.0, 0.0)	0.0 (0.0, 0.1)	0.1 (0.0, 0.2)
*Frequency and Duration (Number of Days)*					
Days > 16°C	Tectah:	0	0	0.9 (0.0, 2.1)	42.9 (31.5, 53.8)
	Lost Man:	0	0	0	0
Consecutive Days > 16°C	Tectah:	0	0	0.5 (0.0, 1.3)	31.1 (21.0, 41.1)
	Lost Man:	0	0	0	0

**Table 3:** Stream thermal regime responses in downstream reaches.

Temperature Response (Δ°C)	Watershed	Fall	Winter	Spring	Summer
*Magnitude*			** **	** **	** **
Daily Maximum	Tectah:	0.6 (0.3, 0.8)	0.0 (-0.2, 0.2)	1.0 (0.3, 1.8)	1.3 (0.7, 2.1)
	Lost Man:	0.1 (0.0, 0.1)	0.1 (0.0, 0.1)	0.1 (-0.1, 0.3)	0.1 (-0.1, 0.3)
MWMT	Tectah:	0.3 (0.0, 0.5)	0.0 (-0.1, 0.1)	0.9 (0.1, 1.6)	1.3 (0.7, 2.1)
	Lost Man:	0.1 (0.0, 0.1)	0.1 (0.0, 0.1)	0.1 (-0.1, 0.2)	0.1 (-0.1, 0.2)
Daily Mean	Tectah:	0.1 (-0.1, 0.1)	0.1 (-0.1, 0.1)	0.1 (-0.1, 0.3)	0.5 (0.2, 0.7)
	Lost Man:	0.0 (-0.1, 0.1)	0.1 (0.0, 0.1)	-0.1 (-0.1, 0.1)	0.0 (-0.1, 0.1)
MWAT	Tectah:	0.1 (-0.1, 0.2)	0.0 (-0.1, 0.1)	0.3 (-0.1, 0.6)	0.6 (0.3, 0.9)
	Lost Man:	0.0 (0.0, 0.1)	0.1 (0.0, 0.1)	0.0 (-0.1, 0.1)	0.0 (-0.1, 0.1)
Degree Days	Tectah:	4.6 (-2.1, 11.8)	1.3 (-1.9, 4.7)	9.6 (-9.3, 23.7)	44.9 (22.0, 68.1)
	Lost Man:	-0.9 (-3.9, 2.2)	4.7 (0.6, 8.9)	-2.3 (-11.6, 7.0)	0.4 (-8.9, 9.8)
Daily Minimum	Tectah:	0.0 (-0.1,0.1)	0.0 (0.0, 0.1)	0.0 (-0.1, 0.1)	0.3 (0.1, 0.4)
	Lost Man:	0.0 (-0.1, 0.0)	0.1 (0.0, 0.1)	-0.1 (-0.1, 0.1)	0.0 (-0.1, 0.1)
*Variability*					
Average Daily Range	Tectah:	0.1 (0.0, 0.2)	0.0 (-0.1, 0.1)	0.3 (-0.1, 0.6)	0.6 (0.2, 1.0)
	Lost Man:	0.0 (0.0, 0.1)	0.0 (0.0, 0.0)	0.0 (-0.1, 0.1)	0.1 (0.0, 0.2)
Maximum Daily Range	Tectah:	0.2 (-0.1, 0.5)	-0.2 (-0.6, 0.3)	0.8 (-0.1, 1.6)	0.7 (-0.1, 1.6)
	Lost Man:	0.1 (0.0, 0.1)	-0.1 (-0.2, 0.0)	-0.4 (-0.9, 0.1)	0.2 (0.1, 0.3)
Average Variance	Tectah:	0.1 0.0 (0.0, 0.1)	0.0 (0.0, 0.0)	0.2 (0.0, 0.4)	0.4 (0.1, 0.8)
	Lost Man:	0.0 (0.0, 0.0)	0.0 (0.0, 0.0)	0.0 (0.0, 0.0)	0.0 (0.0, 0.0)
Maximum Variance	Tectah:	0.1 (0.0, 0.3)	0.0 (-0.1, 0.1)	0.8 (0.0, 1.6)	0.9 (0.1, 1.7)
	Lost Man:	0.0 (0.0, 0.0)	0.0 (-0.1, 0.0)	0.0 (0.0, 0.0)	0.1 (0.0, 0.1)
*Frequency and Duration (Number of Days)*					
Days > 16°C	Tectah:	0	0	0.4 (0.0, 1.1)	16.3 (6.1, 27.4)
	Lost Man:	0	0	0	0
Consecutive Days > 16°C	Tectah:	0	0	0.3 (0.0, 0.8)	11.6 (3.9, 20.0)
	Lost Man:	0	0	0	0

Summary of temperature responses in thinned reaches for selected descriptors of stream thermal regimes including magnitude, variability, frequency, and duration in northern California second-growth redwood forests. Temperature responses are mean estimates of BACI differences for sites within the Tectah and Lost Man watersheds with lower and upper 95% confidence intervals in parentheses. We estimated non-parametric 95% confidence intervals using a bootstrapping protocol if responses did not follow a normal distribution in the boot package in R [56]. Temperature responses were summarized according to four seasons: Fall (October-December), Winter (January-March), Spring (April-June), and Summer (July–September). No pre-treatment data were available during fall and winter seasons for Lost Man so values reflect post-treatment differences between thinned and upstream reaches. See text for explanations of response variable acronyms.

Thinning increased thermal variability in thinned and downstream reaches, but responses varied between seasons and watersheds (Fig 3, Tables 2 and 3). In the Tectah watersheds, BACI models indicated that increases in thermal variability in thinned reaches were most pronounced during summer increasing the daily range by a mean of 2.5°C (95% confidence intervals: 1.6, 3.4) and variance by a mean of 1.6°C (0.7, 2.5), but also increased during spring (daily range: 0.5°C; variance: 0.3°C) and fall (daily range: 0.4°C; variance: 0.1°C) (S1 Table). Increases in thermal variability in downstream reaches were limited to summer (daily range: 0.7°C; variance: 0.5°C) (S1 Table). In the Lost Man watershed, BACI models indicated no effect of thinning on thermal variability in thinned or downstream reaches (S1 Table).

Thinning increased the frequency and duration of warm water events in thinned and downstream reaches, but responses occurred exclusively in the Tectah watersheds (Fig 3, Tables 2 and 3). The frequency of days with temperatures greater than 16°C increased in summer by a mean of 42.9 more days (95% confidence intervals: 31.5, 53.8) in thinned reaches and a mean of 16.3 more days (6.1, 27.4) in downstream reaches (Tables 2 and 3). Temperatures greater than 16°C persisted for a mean duration of 31.1 more consecutive days (21.0, 41.1) in thinned reaches and 11.6 more consecutive days (3.9, 20.0) in downstream reaches (Tables 2 and 3). Responses in frequency and duration occurred earlier in the year, starting in spring in both thinned and downstream reaches (Tables 2 and 3). Temperatures exceeded 20°C in two of the eight sites in the Tectah watersheds. Within these locations, the WFT1_low site temperatures exceeded 20°C for a period of 30 days and 14 consecutive days, while the WFT2_up site exceeded 20°C for a period of 3 days.

Summary of temperature responses in downstream reaches for selected descriptors of stream thermal regimes including magnitude, variability, frequency, and duration in northern California second-growth redwood forests. Temperature responses are mean estimates of BACI differences for sites within the Tectah and Lost Man watersheds with lower and upper 95% confidence intervals in parentheses. We estimated non-parametric 95% confidence intervals using a bootstrapping protocol if responses did not follow a normal distribution in the boot package in R [56]. Temperature responses were summarized according to four seasons: Fall (October-December), Winter (January-March), Spring (April-June), and Summer (July–September). No pre-treatment data were available during fall and winter seasons for Lost Man so values reflect post-treatment differences between downstream and upstream reaches. See text for explanations of response variable acronyms.

### Longitudinal profiles

**Fig 4:**
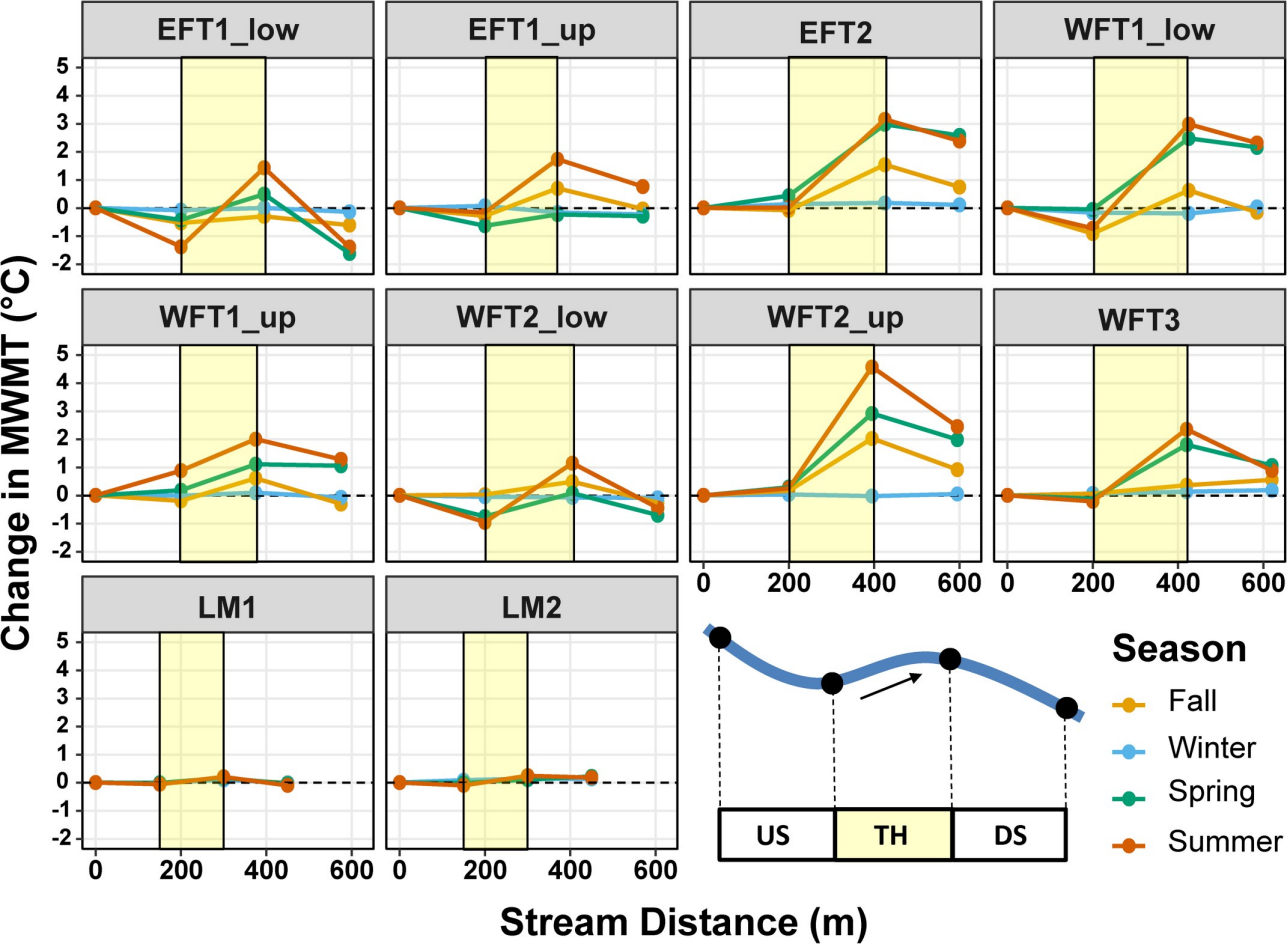
Reach-scale longitudinal profiles of stream temperature responses. Seasonal variation in reach-scale longitudinal profiles of stream temperature responses (pre-treatment—post-treatment) in upstream reference (US), thinned (TH), and downstream (DS) reaches for individual sites (n = 10) in northern California second-growth redwood forests. The position of thinned reaches is indicated by the yellow polygons. Black dots on blue line depict temperature sensor locations along upstream, thinned, and downstream reaches.

Reach-scale longitudinal profiles indicated that local temperature responses associated with thinning frequently persisted into downstream reaches, but the extent of downstream responses reflected the magnitude and timing of local increases (Fig 4). In the Tectah watersheds, local increases in temperature were highest in the summer in all sites, followed by spring and fall, and lowest in the winter (Fig 4). Downstream trajectories varied in direction where in some sites temperatures remained elevated at the downstream extent of the downstream reach (e.g., EFT1_up, EFT2, WFT1_low, WFT1_up, WFT2_up, and WFT3), whereas in other sites local increases recovered to its initial state at the downstream extent of the downstream reach (e.g., EFT1_low, WFT2_low). In contrast, Lost Man sites showed no to minor responses in temperature in thinned or downstream reaches during any of the four seasons (Fig 4).

### Multivariate analyses of thermal regimes

**Fig 5:**
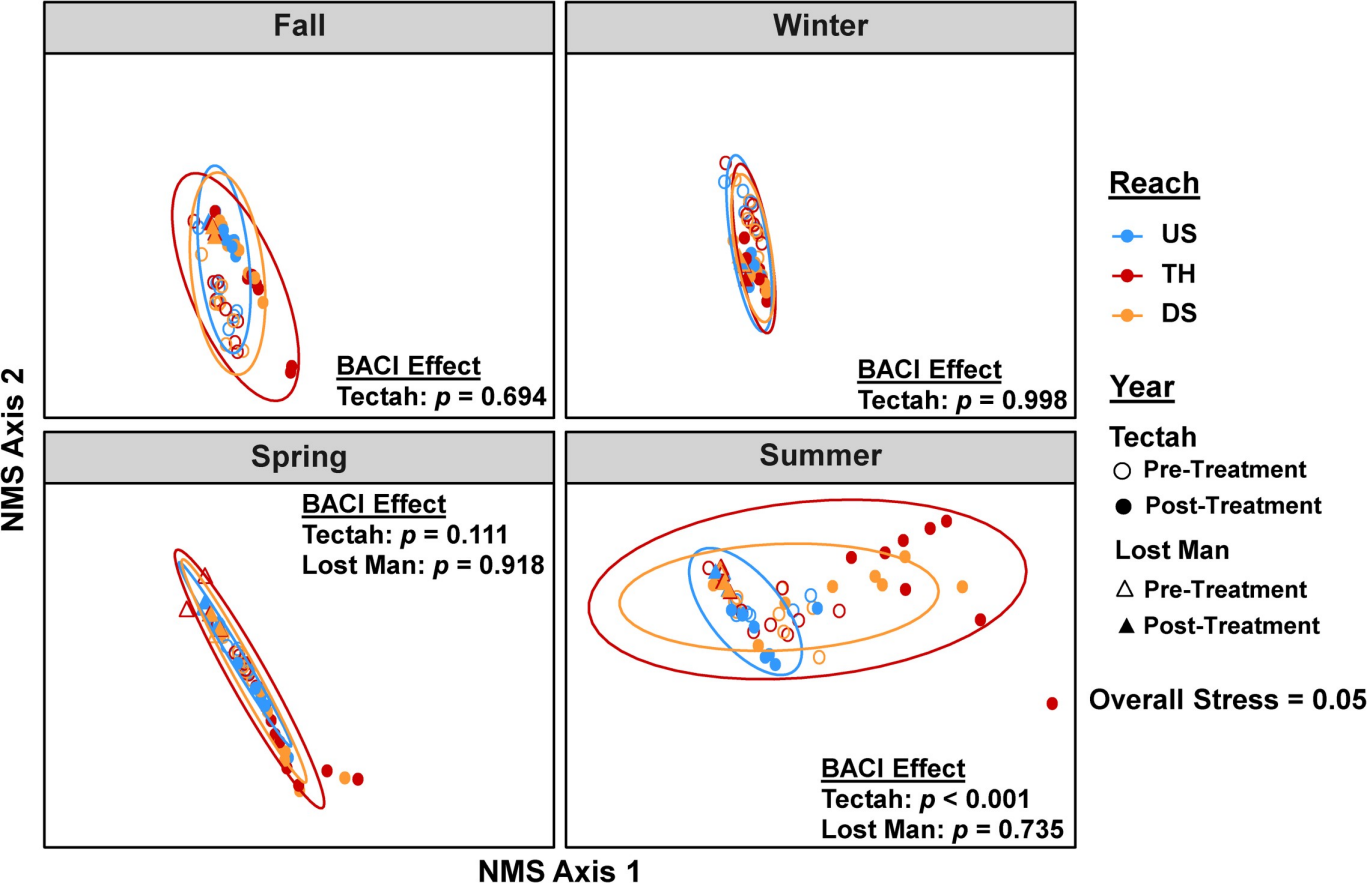
NMS ordinations of stream thermal regimes. Non-metric multidimensional scaling ordinations of the structure of stream thermal regimes in response to riparian thinning in northern California second-growth redwood forests. Each point represents the structure of a stream thermal regime at a site with colors indicating reach type and shapes indicating pre-treatment or post-treatment years for each watershed. The proximity of points provides an indication of how similar thermal regimes are between reaches (upstream reference, thinned, downstream), years (pre-treatment, post-treatment), and seasons (fall, winter, spring, summer) for the Tectah and Lost Man watersheds. Ellipses indicate 95% confidence intervals around the grouping variable of reach. PerMANOVA tests indicated whether the structure of stream thermal regimes differed significantly by the BACI effect of Reach*Year (α = 0.05).

NMS ordinations indicated that thinning shifted the structure of stream thermal regimes in thinned and downstream reaches, but the extent of these shifts varied seasonally and between watersheds (Fig 5). Shifts in the structure of thermal regimes were observed in the Tectah watershed during the post-treatment year in thinned and downstream reaches, but did not change in the Lost Man watershed (Fig 5). Shifts occurred primarily during summer, but were also visible to a lesser extent during fall and spring. No change in the structure of stream thermal regimes was evident during winter. PerMANOVA tests partially supported the patterns in NMS ordinations and documented that the structure of stream thermal regimes differed in the Tectah watersheds in summer, but not in Lost Man during any season (BACI effect: *p* <0.05) (Fig 5).

### Environmental factors

**Fig 6:**
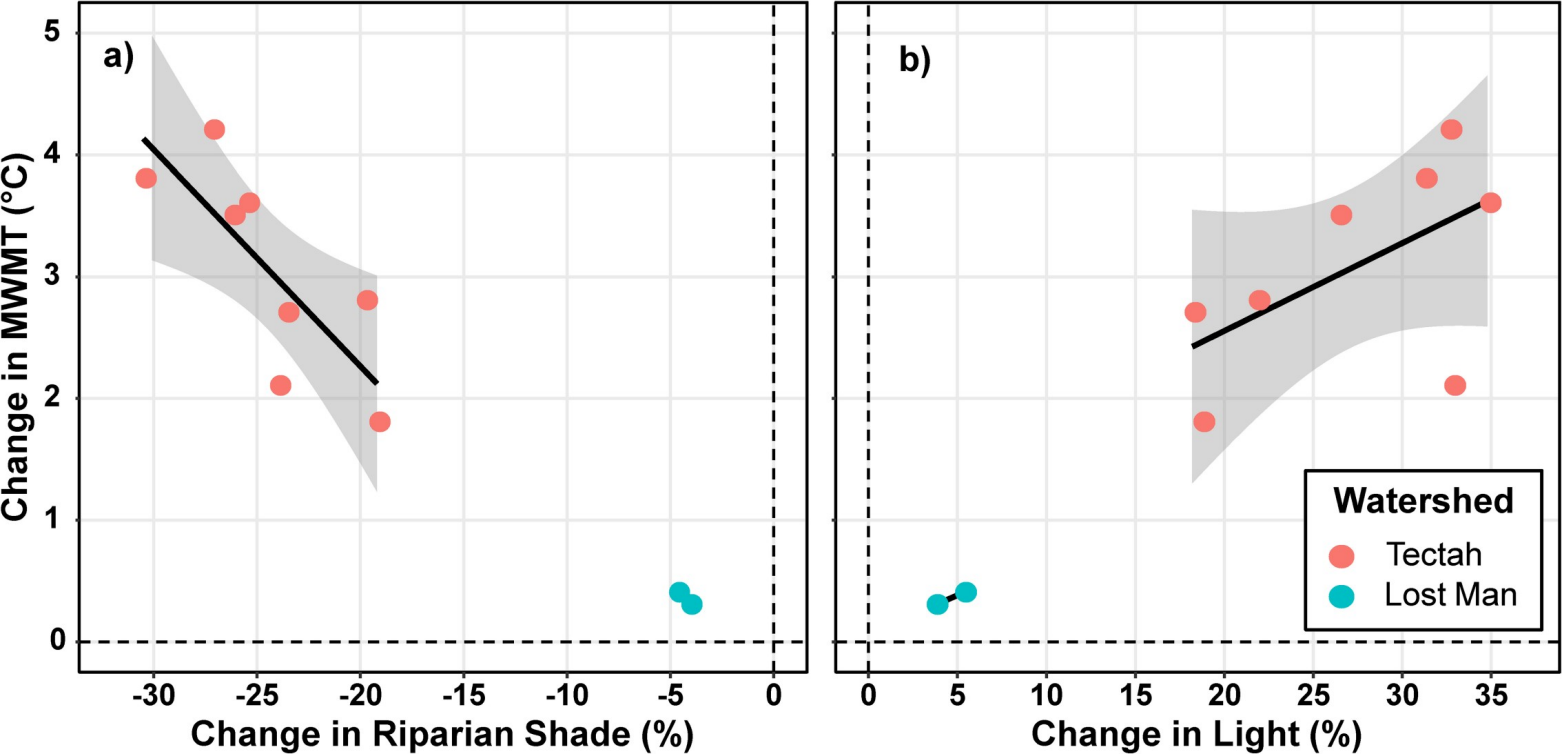
Relationships between shade, light, and stream temperature. Relationships between the responses of riparian shade (a) and light (b) and stream temperature (summer MWMT) associated with riparian thinning treatments in Tectah and Lost Man watersheds in northern California second-growth redwood forests. Responses are calculated as BACI differences. Gray shading indicates 95% confidence intervals.

Across all watersheds, the magnitude of stream temperature responses to thinning were associated with the extent of changes in shade and light (Fig 6). However, the strength of these relationships varied between watersheds. In the Tectah watersheds, summer MWMT correlated strongly with shade (*r* = -0.75) and light (*r* = 0.76) as well as upstream temperatures (*r* = 0.51) and proximity to upstream treatments (*r* = 0.56) (S1 Fig). In the Lost Man watershed, summer MWMT did not correlate with shade or light, but did correlate with air temperature (*r* = 0.91), upstream temperatures (*r* = 0.73) as well as physical site characteristics such distance upstream (*r* = -0.91), bankfull width (*r* = 0.80), gradient (*r* = 0.81), aspect (*r* = -0.77), and elevation (*r* = -0.89), although many of these variables correlated with one another (S1 Fig).

### Model selection

The AIC_c_ model selection process indicated that distinct models explained the variation in thermal conditions in each watershed. In the Tectah watersheds, models that included continuous estimates of riparian shade and upstream thermal conditions consistently ranked at the top of candidate sets for all temperature descriptors considered (S2 Table). In addition to riparian shade and upstream temperatures, proximity to upstream treatments appeared as an important variable for MWAT, degree days, and variance (S2 Table). Gradient appeared in the top models for degree days, daily range, and variance (S2 Table), but otherwise physical variables contributed little toward explaining model variance. In the Lost Man watershed, no single variable consistently ranked at the top of candidate model sets and the null (intercept) model often parsimoniously outperformed other variables in explaining model variation for many descriptors (S2 Table). Exceptions to this were for MWAT and degree days which indicated that gradient and upstream temperatures provided slightly better explanations than the null model (S2 Table). For all watersheds, best supported models ranked substantially higher than the BACI model of Reach*Year, which frequently ranked towards the bottom of the candidate sets of the models we considered (S2 Table).

## Discussion

In this study we found that responses to the experimental riparian thinning treatments we evaluated differed greatly depending on treatment intensity. In the Tectah watersheds where thinning treatments were more intensive the reductions in shade and increases in light were sufficient to shift stream thermal regimes in thinned and downstream reaches. Thinning treatments were less intensive in the Lost Man watershed, resulting in small changes in shade and light that had minimal influence on stream temperatures. These results suggest that riparian thinning can influence thermal conditions of small streams both locally and further downstream, but the extent of the thermal responses depend on the amount of shade lost and light gained.

Riparian shade and light responses to thinning varied strongly between watersheds. Reductions in riparian shade were five to six times more intensive in the Tectah watersheds versus Lost Man and increases in light were nine to ten times more intensive. BACI models supported these patterns indicating significant reductions in shade and increases in light in the Tectah watersheds, yet the less intensive treatments in the Lost Man watershed were only significant as effective shade. Although all thinning treatment prescriptions targeted a 40–50% reduction in canopy closure or basal area, estimates of riparian shade and light indicated much smaller overall, yet more variable changes over the stream channel. This discrepancy could be due to the fact that thinning prescriptions were made from within the riparian forest and not over the stream channel. Alternatively it could be due to differences in prescription methods where targets based on canopy closure resulted in larger changes in shade and light whereas targets based on basal area resulted in smaller changes. Slope restrictions in the steep Lost Man watershed (no thinning on slopes greater than 20%) likely provided additional constraints to the implementation of thinning treatments. Although the thinning treatments included in this study were not consistent across the study watersheds, this range in treatment intensity provided a broad range of conditions for us to evaluate.

Stream temperature responses to thinning reflected the magnitude of changes in shade and light, ranging widely between watersheds. In the Tectah watersheds where treatment intensity was higher, BACI models indicated that thinning treatments altered stream temperatures in thinned and downstream reaches across multiple descriptors of the thermal regime and these responses extended over multiple seasons. In contrast, BACI models indicated that stream temperatures did not change in the Lost Man watershed in thinned or downstream reaches for any descriptor or any season.

Overall, the temperature responses to thinning observed in this study were lower than previously documented responses to historical timber harvest practices which often clearcut forests to the stream edge [4, 24, 25]. Instead our results coincided more closely to stream temperature responses observed with contemporary forest management practices that include riparian buffers which have often resulted in smaller, yet more variable temperature increases [26–29]. Variation in temperature responses in these contemporary studies tends to be associated with riparian buffer width where undetectable to small changes in temperature coincided with wider buffers [26, 27] or riparian buffers of various widths adjacent to upland thinning [14, 57], whereas larger increases in stream temperatures were more likely to occur with narrower buffers adjacent to upland clearcutting [26, 27].

Few comparable analyses of forest thinning within riparian buffers exist in the literature. However, a study by Rex et al. [58] found that variable-retention treatments within riparian buffers in British Columbia that reduced riparian shade between 30 and 50% increased MWAT by 3°C and MWMT by 5–6°C, both higher than documented in our study. Studinski et al. [59] found that thinning treatments that targeted a 50% reduction in basal area in some West Virginia streams resulted in a similar reduction in canopy closure to the treatments in the Tectah watersheds, yet resulted in much smaller increases (0.2–0.5°C/100m) than what we observed and were more in line with the responses documented in Lost Man. Another study in Minnesotan boreal streams found that their most intensive thinning treatment resulted in a 10% reduction in canopy closure but increased summer maximum temperatures by ~4°C [60]. These studies highlight that the magnitude of responses to thinning are often system dependent, making broader-scale generalizations challenging.

Local temperature responses to thinning were not limited to thinned reaches and effects frequently extended into downstream reaches. Downstream effects reflected the magnitude and timing of upstream temperature increases and were typically ~50% of the response observed in respective thinned reaches, similar to results observed by Davis et al. [61] ~300 m downstream of harvest. Longitudinal profiles revealed three distinct downstream trajectories at the reach scale, with temperature remaining elevated 150 to 200 m downstream, dissipating either partially or completely, or remaining undetectable where minimal change occurred upstream (e.g., Lost Man). Downstream effects sometimes propagated beyond the extent of the downstream reach and into adjacent sites where sequentially located. Subsequent temperature responses were more likely to be elevated, which suggests the potential for cumulative heating in cases where harvests are spaced closer together. These patterns suggest that local temperature within our sites were not independent from upstream sites and that there was a high degree of longitudinal connectivity in these streams [4, 62]. Although we limited our analysis to immediate reach-scale responses in downstream effects ~150–200 m downstream from thinning treatments, we recognize that in some reaches the spatial extent of downstream effects likely extended further [52, 61]. For example, Wilzbach et al. [63] documented that local increases in temperature associated with complete canopy removal along a 100 m reach persisted up to 430 m downstream.

Thermal responses to thinning exhibited strong seasonal variation, although the extent of seasonal dependence varied between watersheds. For all watersheds, temperature responses were greatest in summer, which coincided with the period of low flows in these watersheds [64]. However, in the Tectah watersheds where thinning treatments were more intensive, thermal responses extended beyond summer into fall and the following spring, consistent with findings from Washington State [65]. In the Tectah watersheds, multi-seasonal responses were most evident for MWMT, daily range and variance, whereas responses of other descriptors and downstream reaches were limited to summer months. Temperature exceedances over common cold-water thresholds were primarily limited to summer months similar to patterns observed in McIntyre et al. [65], although a few sites also exceeded 16°C in the spring. We did not observe treatment effects in the winter. Winters in these coastal systems are characterized by high flows and weak solar radiation filtered by dense clouds and coastal fog, which would likely limit the influence of any differences in canopy conditions associated with thinning [23]. Most temperature studies focus their analyses on summer conditions and so few attempt to quantify the seasonality of thermal responses. By collecting year-round data we successfully tracked not just the magnitude of thermal responses to thinning, but also the timing and temporal duration of those changes. Given that thermal regimes naturally fluctuate seasonally in the Pacific Northwest [57], a better understanding of the timing and temporal duration of these changes provide important information for managers when considering the effects on sensitive aquatic species.

Our multivariate analyses effectively captured the multidimensional local and downstream shifts in the structure of stream thermal regimes due to thinning. Similar to other analyses, multivariate responses varied between watersheds. In the Lost Man watershed, we detected no structural changes in stream thermal regimes. However, in the Tectah watersheds we observed that shifts in thermal regimes within thinned and downstream reaches that peaked in summer, but also were observed to a lesser extent during spring and fall, whereas all sites were similar in winter. Our results align with recent efforts to better characterize thermal regimes [31, 33] and how they may shift to disturbances such as wildfire [66]. These studies highlight that thermal responses to disturbance are not limited to single descriptors (e.g., summer maxima) but can shift in multiple directions. Although specific descriptors of magnitude such MWMT and MWAT are important for regulatory purposes [29, 34], other descriptors such as changes in thermal variability and the frequency and duration of those changes may have more relevance for affecting ecological processes and aquatic species in streams [30, 47]. A thermal regime approach as applied here provided additional value as a more holistic evaluation of overall thermal changes not possible by relying on individual descriptors alone [32, 33].

Analyses that further explored the environmental factors driving the variation in stream thermal regimes and their responses to thinning observed distinct sets of drivers in each watershed. In the Tectah watersheds, variation in thermal responses appear to be largely driven by reductions in riparian shade and increases in light. Correlation analyses revealed that the intensity of temperature responses in thinned reaches was strongly associated with the amount of shade lost and light gained following the thinning treatments. Model selection analyses supported this pattern finding that including continuous estimates of shade more effectively captured the variation in thermal responses that the categorical variables in the BACI model could not. These results align with the findings of Johnson [67] and Cassie [23] that solar radiation is a primary driver of energy budgets in small streams. In contrast neither shade nor light were primary drivers of temperature responses in the Lost Man watershed. This is likely because thinning treatments did not increase solar radiation enough to affect stream temperatures. Alternatively, the pervasive groundwater and hyporheic flow in this watershed could have mediated the influence of any increased solar radiation from thinning treatments on stream temperatures.

In addition to local changes in shade and light, our model selection results indicated that upstream thermal conditions and proximity to upstream treatments explained additional variation in thermal responses in the Tectah watersheds. These results suggest that thermal responses depended on both longitudinal advective processes as well as local radiative processes of heat transfer [4]. Groom et al. [26] also documented that upstream thermal conditions act as an important driver of thermal responses to contemporary forest management practices in western Oregon. Our correlation analyses corroborated these results, finding that local temperature response in thinned reaches strongly correlated with upstream temperatures and proximity to upstream treatments. Best supported models sometimes included physical site characteristics such as gradient and reach length, but this was not consistent for all descriptors. Inclusion of these variables suggests that the physical dimensions of study sites only sometimes helped explain thermal responses to thinning, a finding not supported by other stream temperature studies [28].

Although the correlation analysis for the Lost Man watershed indicated that stream temperature correlated with multiple covariates including upstream temperatures and physical site characteristics, the results from the model selection suggests that these covariates poorly explained the variation in stream thermal conditions in this watershed. Our model selection analysis found the null (intercept) model to be the best explanation for multiple temperature descriptors. Although other covariates such upstream temperatures and gradient ranked as the best supported model for two descriptors, they barely outperformed the null model. These results suggest that either the covariates we considered poorly represented the thermal conditions in the Lost Man watershed or that thermal conditions in this watershed did not vary much on their own and so there was little variation to model.

### Management implications

Riparian forests in the Pacific Northwest have been extensively altered by past timber harvest practices and managers now face the challenge of restoring the desirable ecological functions that riparian forests provide as they continue to recover [68]. Managers have leaned towards passive strategies in the past, but these strategies can take centuries to work [69]. As a result, there is growing interest in the application of active management approaches like thinning to help accelerate the recovery of these forests [12, 13]. However, trade-offs can emerge between the long-term benefits of restoring riparian forests and the potential short-term impacts to streams. From an adaptive management perspective, experimental data provide unique information for resource managers to address existing knowledge gaps surrounding the effects of partial canopy removal associated with thinning [70]. We believe our study offers useful insight to managers interested in thinning as a riparian restoration tool. However, a more comprehensive understanding of riparian thinning’s effectiveness will also require additional research.

First, we observed that changes in shade of 5% or less caused minimal changes in temperature while reductions in shade of 20–30% resulted in much larger increases in temperature. Therefore, managers could set thinning prescriptions to strike a balance between minimizing increases in temperature while also achieving riparian restoration objectives. Future studies could examine thinning treatments at a more comprehensive range of intensities, including more intermediate intensities, to help determine how much forests can be thinned without impacting stream temperatures.

Second, we observed that thinning increased downstream temperatures and that incoming thermal conditions and proximity to upstream treatments helped explain the variation of the temperature responses. Therefore, it is important to consider the longitudinal spacing between treatments to control absolute stream temperatures. Further research needs to establish how far downstream temperature responses can travel [61]. This information would be useful for developing treatment spacing guidelines for avoiding potential cumulative effects [71].

Third, we only evaluated immediate responses one year post-treatment and questions remain about the duration of these responses as forests recover over time. Other studies have found that post-treatment increases in temperature often peak one to two years post-treatment [48, 52]. Future research needs to monitor the length of time necessary for the riparian canopy to fill in and for stream temperatures to recover.

Fourth, this study was conducted in three small coastal streams (watershed areas <10 km^2^ all within 15 km of the Pacific Ocean), and as a result our scope of inference is limited to the study watersheds, but may also apply to systems with comparable characteristics. More examples of riparian thinning are needed from a wider range of stream sizes, underlying lithologies, flow regimes, geomorphologies, climates, and other factors that can lead to the context dependency so frequently observed in stream temperature studies. Observations from more locations under a broader range of conditions would improve an understanding of the intensity and spatial frequency of riparian thinning for achieving restoration goals for second-growth riparian forests.

Finally, the changes in stream temperature documented here have broad, complex ecological implications for the aquatic species that occupy these watersheds [22]. Future studies could establish the lethal and sublethal effects that the thermal responses observed here may have on cold-water adapted species such as stream fish or amphibians [39, 40].

## Conclusions

From the experimental riparian thinning treatments evaluated in this study, we found that the responses to thinning ranged widely depending on the intensity of treatment. In the Tectah watersheds where thinning treatments were more intensive, reductions in shade and increases in light were sufficient to shift stream thermal regimes locally and in downstream reaches across multiple seasons. However, in the Lost Man watershed where thinning treatments were less intensive, small changes in shade and light resulted in minimal changes to stream temperatures. These results suggest that thinning within riparian zones in second-growth redwood forests may be a feasible restoration strategy without impacting stream temperatures when conducted less intensively. Collectively, this study provides new insights into the effects of riparian thinning on reach-scale responses of shade, light, and stream thermal regimes. The results from this study provide relevant information for managers to help guide decisions about whether and how much thinning may be applied to restore second-growth riparian forests recovering from previous harvest.

## Supporting information

S1 Fig Correlation matrix for Tectah and Lost Man watersheds. (DOCX)

S1 Table Summary of BACI analyses. (DOCX)

S2 Table Model selection AIC_c_ table ranking all *a priori* candidate models. (DOCX)

S3 Table Best supported models determined by model selection. (DOCX)

S1 File Reference sites analysis. (PDF)
