# Correction: Shade, light, and stream temperature responses to riparian thinning in second-growth redwood forests of northern California

**DOI:** 10.1371/journal.pone.0314295

**Published:** 2024-11-18

**Authors:** The PLOS One Staff

The Table footnotes for Tables 2 and 3 were formatted incorrectly and placed in the main body of the text of the results section. The text for one of the table footnotes (Table 2) occurs directly below the table in the main text, but the other (Table 3) occurs two paragraphs below in the main body of the text. Please see the correct Tables 2 and 3 and its footnotes here.

**Table 2:** Stream thermal regime responses in thinned reaches.

Temperature Response (Δ°C)	Watershed	Fall	Winter	Spring	Summer
*Magnitude*					
Daily Maximum	Tectah: Lost Man:	1.4 (1.0, 1.8) 0.1 (0.1, 0.2)	0.1 (-0.1, 0.2) 0.1 (0.0, 0.1)	1.7 (1.1, 2.3) 0.4 (0.1, 0.6)	2.9 (2.1, 3.6) 0.3 (0.1, 0.5)
MWMT	Tectah: Lost Man:	1.0 (0.6, 1.4) 0.1 (-0.1, 0.1)	-0.1 (-0.1, 0.1) 0.1 (0.0, 0.1)	1.6 (1.0, 2.2) 0.1 (-0.2, 0.5)	2.7 (2.0, 3.4) 0.2 (-0.1, 0.4)
Daily Mean	Tectah: Lost Man:	0.0 (-0.1, 0.1) 0.1 (0.0, 0.1)	0.0 (-0.1, 0.1) 0.1 (0.0, 0.1)	0.2 (0.1, 0.3) -0.2 (-0.6, 0.1)	0.8 (0.6, 1.0) 0.2 (0.1, 0.2)
MWAT	Tectah: Lost Man:	0.1 (0.0, 0.2) 0.1 (0.0, 0.1)	0.0 (-0.1, 0.1) 0.1 (0.0, 0.1)	0.5 (0.3, 0.6) 0.0 (-0.2, 0.1)	0.9 (0.6, 1.1) 0.2 (0.1, 0.3)
Degree Days	Tectah: Lost Man:	3.3 (-3.0, 9.6) 5.6 (-0.7, 11.9)	0.7 (-2.2, 3.5) 5.8 (2.7, 8.9)	17.1 (8.7, 23.4) 6.8 (6.1, 7.5)	73.4 (56.5, 90.3) 13.8 (9.5, 18.1)
Daily Minimum	Tectah: Lost Man:	-0.1 (-0.2, 0.0) 0.0 (-0.1, 0.1)	0.0 (-0.1, 0.0) 0.1 (0.0, 0.1)	0.0 (0.0, 0.1) -0.1 (-0.2, 0.1)	0.1 (-0.1, 0.3) 0.1 (0.0, 0.1)
*Variability*					
Average Daily Range	Tectah: Lost Man:	0.4 (0.2, 0.5) 0.0 (0.0, 0.1)	0.0 (-0.1, 0.1) 0.0 (0.0, 0.1)	0.5 (0.3, 0.8) 0.2 (0.0, 0.4)	2.4 (1.8, 3.0) 0.1 (0.0, 0.1)
Maximum Daily Range	Tectah: Lost Man:	1.2 (0.7, 1.8) 0.0 (0.0, 0.0)	-0.1 (-0.4, 0.2) 0.1 (0.0, 0.3)	1.5 (0.7, 2.2) 0.2 (0.1, 0.2)	2.9 (2.0, 3.8) 0.1 (0.1, 0.2)
Average Variance	Tectah: Lost Man:	0.1 (0.1, 0.2) 0.0 (0.0, 0.0)	0.0 (-0.1, 0.1) 0.0 (0.0, 0.0)	0.3 (0.1, 0.5) 0.0 (0.0, 0.0)	1.5 (0.9, 2.3) 0.0 (0.0, 0.1)
Maximum Variance	Tectah: Lost Man:	0.7 (0.3, 1.2) 0.0 (0.0, 0.1)	-0.1 (-0.1, 0.1) 0.0 (0.0, 0.0)	1.2 (0.5, 2.0) 0.0 (0.0, 0.1)	2.4 (1.4, 3.7) 0.1 (0.0, 0.2)
*Frequency and Duration (Number of Days)*					
Days > 16°C	Tectah: Lost Man:	0 0	0 0	0.9 (0.0, 2.1) 0	42.9 (31.5, 53.8) 0
Consecutive Days > 16°C	Tectah: Lost Man:	0 0	0 0	0.5 (0.0, 1.3) 0	31.1 (21.0, 41.1) 0

Summary of temperature responses in thinned reaches for selected descriptors of stream thermal regimes including magnitude, variability, frequency, and duration in northern California second-growth redwood forests. Temperature responses are mean estimates of BACI differences for sites within the Tectah and Lost Man watersheds with lower and upper 95% confidence intervals in parentheses. We estimated non-parametric 95% confidence intervals using a bootstrapping protocol if responses did not follow a normal distribution in the boot package in R [56]. Temperature responses were summarized according to four seasons: Fall (October-December), Winter (January-March), Spring (April-June), and Summer (July–September). No pre-treatment data were available during fall and winter seasons for Lost Man so values reflect post-treatment differences between thinned and upstream reaches. See text for explanations of response variable acronyms.

**Table 3:** Stream thermal regime responses in downstream reaches.

Temperature Response (Δ°C)	Watershed	Fall	Winter	Spring	Summer
*Magnitude*					
Daily Maximum	Tectah: Lost Man:	0.6 (0.3, 0.8) 0.1 (0.0, 0.1)	0.0 (-0.2, 0.2) 0.1 (0.0, 0.1)	1.0 (0.3, 1.8) 0.1 (-0.1, 0.3)	1.3 (0.7, 2.1) 0.1 (-0.1, 0.3)
MWMT	Tectah: Lost Man:	0.3 (0.0, 0.5) 0.1 (0.0, 0.1)	0.0 (-0.1, 0.1) 0.1 (0.0, 0.1)	0.9 (0.1, 1.6) 0.1 (-0.1, 0.2)	1.3 (0.7, 2.1) 0.1 (-0.1, 0.2)
Daily Mean	Tectah: Lost Man:	0.1 (-0.1, 0.1) 0.0 (-0.1, 0.1)	0.1 (-0.1, 0.1) 0.1 (0.0, 0.1)	0.1 (-0.1, 0.3) -0.1 (-0.1, 0.1)	0.5 (0.2, 0.7) 0.0 (-0.1, 0.1)
MWAT	Tectah: Lost Man:	0.1 (-0.1, 0.2) 0.0 (0.0, 0.1)	0.0 (-0.1, 0.1) 0.1 (0.0, 0.1)	0.3 (-0.1, 0.6) 0.0 (-0.1, 0.1)	0.6 (0.3, 0.9) 0.0 (-0.1, 0.1)
Degree Days	Tectah: Lost Man:	4.6 (-2.1, 11.8) -0.9 (-3.9, 2.2)	1.3 (-1.9, 4.7) 4.7 (0.6, 8.9)	9.6 (-9.3, 23.7) -2.3 (-11.6, 7.0)	44.9 (22.0, 68.1) 0.4 (-8.9, 9.8)
Daily Minimum	Tectah: Lost Man:	0.0 (-0.1, 0.1) 0.0 (-0.1, 0.0)	0.0 (0.0, 0.1) 0.1 (0.0, 0.1)	0.0 (-0.1, 0.1) -0.1 (-0.1, 0.1)	0.3 (0.1, 0.4) 0.0 (-0.1, 0.1)
*Variability*					
Average Daily Range	Tectah: Lost Man:	0.1 (0.0, 0.2) 0.0 (0.0, 0.1)	0.0 (-0.1, 0.1) 0.0 (0.0, 0.0)	0.3 (-0.1, 0.6) 0.0 (-0.1, 0.1)	0.6 (0.2, 1.0) 0.1 (0.0, 0.2)
Maximum Daily Range	Tectah: Lost Man:	0.2 (-0.1, 0.5) 0.1 (0.0, 0.1)	-0.2 (-0.6, 0.3) -0.1 (-0.2, 0.0)	0.8 (-0.1, 1.6) -0.4 (-0.9, 0.1)	0.7 (-0.1, 1.6) 0.2 (0.1, 0.3)
Average Variance	Tectah: Lost Man:	0.0 (0.0, 0.1) 0.0 (0.0, 0.0)	0.0 (0.0, 0.0) 0.0 (0.0, 0.0)	0.2 (0.0, 0.4) 0.0 (0.0, 0.0)	0.4 (0.1, 0.8) 0.0 (0.0, 0.0)
Maximum Variance	Tectah: Lost Man:	0.1 (0.0, 0.3) 0.0 (0.0, 0.0)	0.0 (-0.1, 0.1) 0.0 (-0.1, 0.0)	0.8 (0.0, 1.6) 0.0 (0.0, 0.0)	0.9 (0.1, 1.7) 0.1 (0.0, 0.1)
*Frequency and Duration (Number of Days)*					
Days > 16°C	Tectah: Lost Man:	0 0	0 0	0.4 (0.0, 1.1) 0	16.3 (6.1, 27.4) 0
Consecutive Days > 16°C	Tectah: Lost Man:	0 0	0 0	0.3 (0.0, 0.8) 0	11.6 (3.9, 20.0) 0

Summary of temperature responses in downstream reaches for selected descriptors of stream thermal regimes including magnitude, variability, frequency, and duration in northern California second-growth redwood forests. Temperature responses are mean estimates of BACI differences for sites within the Tectah and Lost Man watersheds with lower and upper 95% confidence intervals in parentheses. We estimated non-parametric 95% confidence intervals using a bootstrapping protocol if responses did not follow a normal distribution in the boot package in R [56]. Temperature responses were summarized according to four seasons: Fall (October-December), Winter (January-March), Spring (April-June), and Summer (July–September). No pre-treatment data were available during fall and winter seasons for Lost Man so values reflect post-treatment differences between downstream and upstream reaches. See text for explanations of response variable acronyms.

The publisher apologizes for the error.
