# Supplementary material for: Shade, light, and stream temperature responses to riparian thinning in second-growth redwood forests of northern California
Source: PLoS One. 2021 Feb 16;16(2):e0246822. doi: 10.1371/journal.pone.0246822 (PMC7886199; doi:10.1371/journal.pone.0246822)
Supplement: S1 Fig — (DOCX) [file pone.0246822.s001.docx]

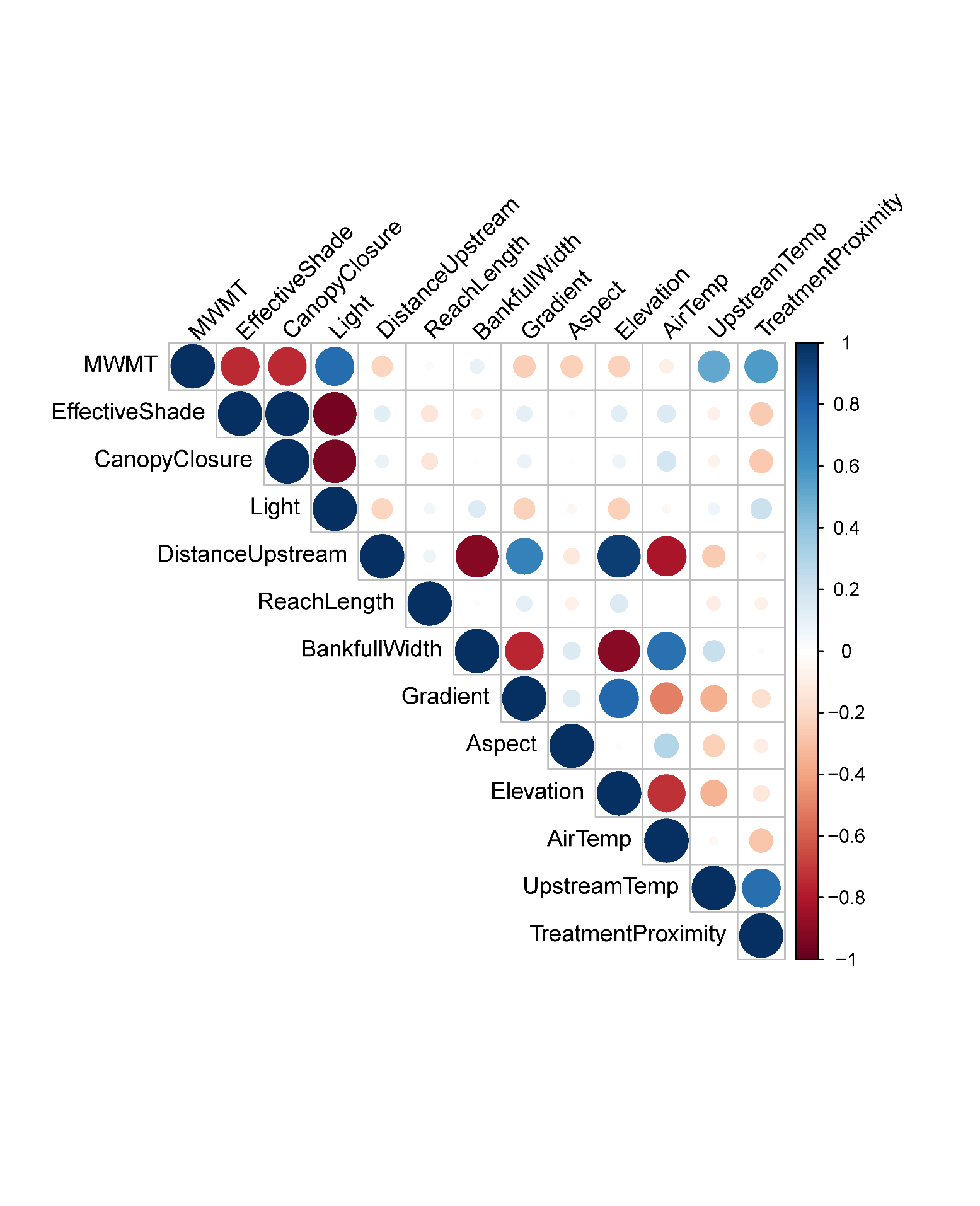


**S1 Fig - a. Correlation matrix for sites in Tectah watersheds.** Environmental drivers of temperature responses for upstream reference, thinned, and downstream reaches using pre-treatment and post-treatment data. Correlation matrix between response in summer MWMT, shade and light responses associated with riparian thinning treatments, and study site characteristics for sites in the Tectah watersheds. Site characteristics included: distance upstream, reach length, bankfull width, aspect, gradient, elevation, air temperature, incoming upstream temperature, landownership, and proximity to upstream treatments.


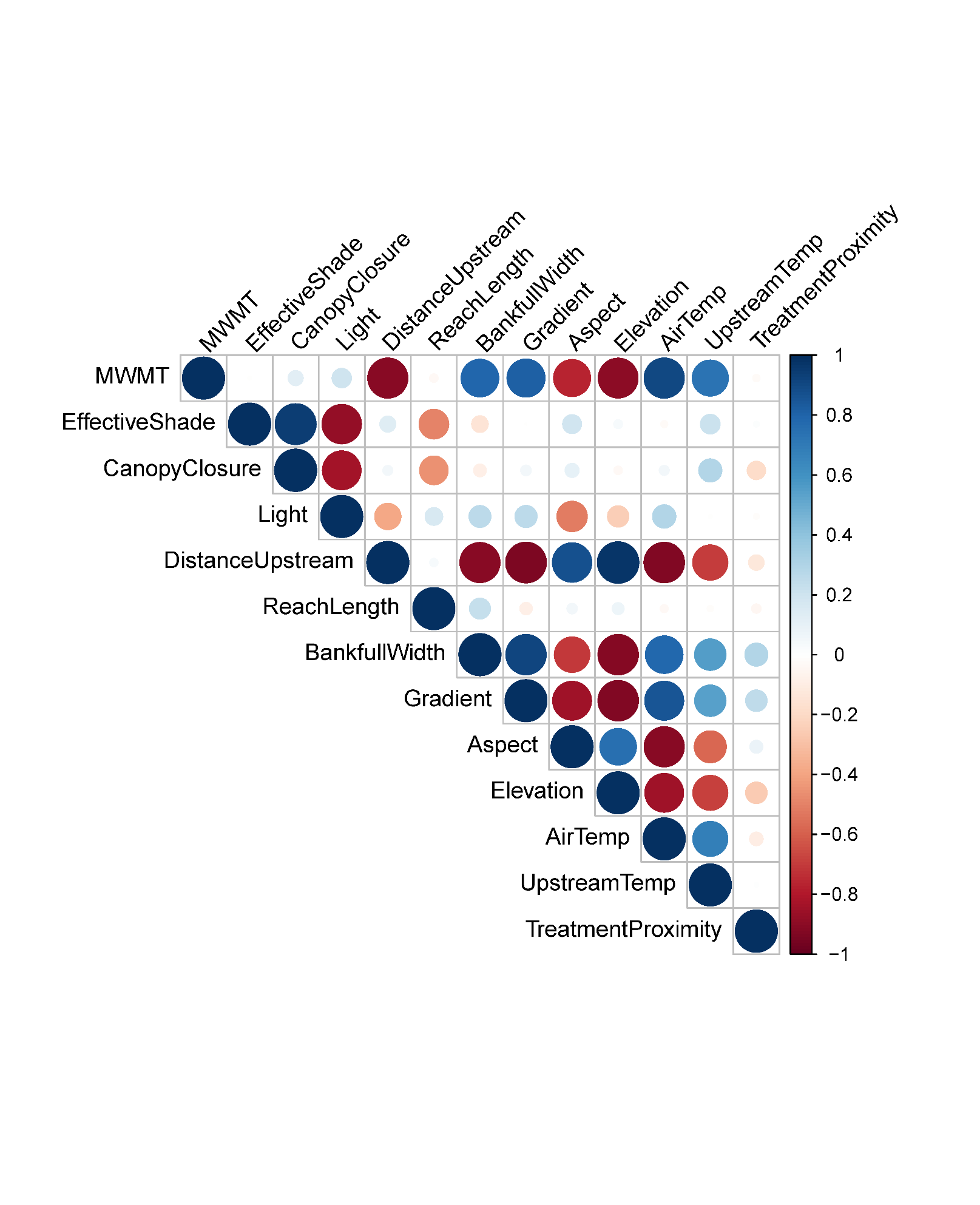


**S1 Fig - b. Correlation matrix for sites in Lost Man watershed.** Environmental drivers of temperature responses for upstream reference, thinned, and downstream reaches using pre-treatment and post-treatment data. Correlation matrix between response in summer MWMT, shade and light responses associated with riparian thinning treatments, and study site characteristics for sites in the Lost Man watersheds. Site characteristics included: distance upstream, reach length, bankfull width, aspect, gradient, elevation, air temperature, incoming upstream temperature, landownership, and proximity to upstream treatments.
