# Supplementary material for: Shade, light, and stream temperature responses to riparian thinning in second-growth redwood forests of northern California
Source: PLoS One. 2021 Feb 16;16(2):e0246822. doi: 10.1371/journal.pone.0246822 (PMC7886199; doi:10.1371/journal.pone.0246822)
Supplement: S1 Table — (DOCX) [file pone.0246822.s002.docx]

**S1 Table. Summary of BACI Analyses.** Summary of BACI models and estimated BACI differences with lower and upper 95% confidence intervals. BACI differences for thinned and downstream reaches are indicated as statistically significant (p < 0.05) by bold font. No pre-treatment stream temperature data were available during fall and winter seasons for Lost Man, so BACI analyses focused on spring and summer. Abbreviations: num DF = numerator degrees of freedom; den DF = denominator degrees of freedom; TH = Thinned; US = Upstream; DS = Downstream.

**BACI model BACI differences – Estimate (Lower 95% CI, Upper 95% CI)**

**Response Variable num DF den DF F-statistic p-value Post-hoc test Post-treatment (2017) Post-treatment (2018)**

*Canopy Closure - Tectah*

Intercept 1 53 6226.8 <0.0001 TH vs. US **-18.7 (-21.0, -16.3)**   **-16.9 (-19.2, -14.6)**

Reach 2 53 0.4 0.6758 DS vs. US 0.6 (-0.7, 1.9) 0.5 (-1.1, 2.1)

Year 2 53 612.0 <0.0001

Reach*Year 4 53 142.5 **<0.0001**

*Canopy Closure - Lost Man*

Intercept 1 8 12030.4 <0.0001 TH vs. US -2.1 (-5.1, 1.4) -1.9 (-5.3, 1.2)

Reach 2 8 19.6 0.0008 DS vs. US 0.5 (-2.9, 3.9) -1.5 (-4.9, 1.9)

Year 2 8 130.4 <0.0001

Reach*Year 4 8 2.7 0.1046

*Effective Shade - Tectah*

Intercept 1 53 1074.4 <0.0001 TH vs. US **-25.4 (-28.6, -22.3) -23.0 (-25.8, -20.1)**

Reach 2 53 40.7 <0.0001 DS vs. US -0.9 (-2.3, 0.5) -1.0 (-2.6, 0.6)

Year 2 53 331.9 <0.0001

Reach*Year 4 53 139.7 **<0.0001**

*Effective Shade - Lost Man*

Intercept 1 8 30329.8 <0.0001 TH vs. US **-4.8 (-8.0, -0.5) -4.1 (-7.3, -0.3)**

Reach 2 8 28.6 0.0002 DS vs. US -0.6 (-2.9, 3.9) -0.5 (-5.1, 4.0)

Year 2 8 250.2 <0.0001

Reach*Year 4 8 4.7  **0.0300**

*Light - Tectah*

Intercept 1 44 422.9 <0.0001 TH vs. US **33.0 (27.4, 38.5) 27.1 (20.4, 33.8)**

Reach 2 44 244.7 <0.0001 DS vs. US 3.2 (-1.6, 4.8) 1.8 (-0.8, 3.9)

Year 2 44 63.3 <0.0001

Reach*Year 4 44 45.3 **<0.0001**

*Light - Lost Man*

Intercept 1 8 496.7 <0.0001 TH vs. US 2.9 (-0.7, 6.5) 2.5 (-1.6, 5.6)

Reach 2 8 13.7 0.0026 DS vs. US -0.4 (-3.3, 2.5) 1.0 (-1.9, 3.9)

Year 2 8 14.6 0.0021

Reach*Year 4 8 1.5 0.2772

**S1 Table continued.**

**BACI model BACI differences – Estimate (Lower 95% CI, Upper 95% CI)**

**Response Variable num DF den DF F-statistic p-value Post-hoc test Post-treatment (2017) Post-treatment (2018)**

*Fall MWMT - Tectah*

Intercept 1 33 26104.8 <0.0001 TH vs. US **1.0 (0.5, 1.5)**

Reach 2 33 11.8 0.0001 DS vs. US 0.3 (-0.1, 0.6)

Year 1 33 69.9 <0.0001

Reach*Year 2 33 7.8  **0.0016**

*Winter MWMT - Tectah*

Intercept 1 33 191084.3 <0.0001 TH vs. US -0.1 (-0.2, 0.1)

Reach 2 33 2.9 0.0672 DS vs. US -0.1 (-0.2, 0.1)

Year 1 33 44.1 <0.0001

Reach*Year 2 33 0.3 0.7273

*Spring MWMT - Tectah*

Intercept 1 33 22810.1 <0.0001 TH vs. US 1**.7 (0.9, 2.5)**

Reach 2 33 3.4 0.0456 DS vs. US **1.0 (0.0, 2.0)**

Year 1 33 0.4 0.5233

Reach*Year 2 33 9.9  **0.0004**

*Summer MWMT - Tectah*

Intercept 1 33 5255.8 <0.0001 TH vs. US **2.8 (1.8, 3.8)**

Reach 2 33 0.5 0.6151 DS vs. US **1.4 (0.3, 2.6)**

Year 1 33 37.4 <0.0001

Reach*Year 2 33 15.6 **<0.0001**

*Spring MWMT – Lost Man*

Intercept 1 5 4440.2 <0.0001 TH vs. US 0.1 (-0.2, 0.5)

Reach 2 5 4.0 0.0919 DS vs. US 0.1 (-0.2, 0.4)

Year 1 5 243.6 <0.0001

Reach*Year 2 5 0.7 0.5209

*Summer MWMT - Lost Man*

Intercept 1 5 6957.8 <0.0001 TH vs. US 0.2 (-0.1, 0.4)

Reach 2 5 3.2 0.1252 DS vs. US 0.1 (-0.2, 0.3)

Year 1 5 7.4 0.0414

Reach*Year 2 5 2.1 0.2216

**S1 Table continued.**

**BACI model BACI differences – Estimate (Lower 95% CI, Upper 95% CI)**

**Response Variable num DF den DF F-statistic p-value Post-hoc test Post-treatment (2017) Post-treatment (2018)**

*Fall MWAT - Tectah*

Intercept 1 33 40736.8 <0.0001 TH vs. US 0.1 (-0.1, 0.3)

Reach 2 33 449.5 <0.0001 DS vs. US 0.1 (-0.1, 0.3)

Year 1 33 362.7 <0.0001

Reach*Year 2 33 0.7 0.5093

*Winter MWAT - Tectah*

Intercept 1 33 82065.7 <0.0001 TH vs. US 0.0 (-0.1, 0.1)

Reach 2 33 0.1 0.9868 DS vs. US 0.0 (-0.1, 0.1)

Year 1 33 31.1 <0.0001

Reach*Year 2 33 0.1 0.9898

*Spring MWAT - Tectah*

Intercept 1 33 58402.4 <0.0001 TH vs. US **0.5 (0.2, 0.8)**

Reach 2 33 15.2 <0.0001 DS vs. US 0.3 (-0.1, 0.7)

Year 1 33 124.5 <0.0001

Reach*Year 2 33 7.4  **0.0022**

*Summer MWAT - Tectah*

Intercept 1 33 24385.0 <0.0001 TH vs. US **0.9 (0.4, 1.4)**

Reach 2 33 0.8 0.4600 DS vs. US **0.6 (0.1, 1.2)**

Year 1 33 55.1 <0.0001

Reach*Year 2 33 7.6  **0.0020**

*Spring MWAT – Lost Man*

Intercept 1 5 13028.6 <0.0001 TH vs. US -0.1 (-0.4, 0.3)

Reach 2 5 1.7 0.2770 DS vs. US 0.0 (-0.3, 0.4)

Year 1 5 297.9 <0.0001

Reach*Year 2 5 0.0 0.9859

*Summer MWAT - Lost Man*

Intercept 1 5 25233.9 <0.0001 TH vs. US 0.1 (-0.1, 0.3)

Reach 2 5 2.5 0.1741 DS vs. US 0.0 (-0.1, 0.2)

Year 1 5 19.0 0.0073

Reach*Year 2 5 3.4 0.1181

**S1 Table continued.**

**BACI model BACI differences – Estimate (Lower 95% CI, Upper 95% CI)**

**Response Variable num DF den DF F-statistic p-value Post-hoc test Post-treatment (2017) Post-treatment (2018)**

*Fall Degree Days - Tectah*

Intercept 1 33 15969.7 <0.0001 TH vs. US 2.9 (-9.1, 14.9)

Reach 2 33 21.0 <0.0001 DS vs. US 5.6 (-6.2, 17.4)

Year 1 33 281.0 <0.0001

Reach*Year 2 33 0.5 0.6025

*Winter Degree Days - Tectah*

Intercept 1 33 21116.7 <0.0001 TH vs. US 0.5 (-5.8, 6.8)

Reach 2 33 0.2 0.7913 DS vs. US 1.1 (-6.4, 8.7)

Year 1 33 3561.0 <0.0001

Reach*Year 2 33 0.1 0.9525

*Spring Degree Days - Tectah*

Intercept 1 33 113137.8 <0.0001 TH vs. US **18.6 (7.5, 29.7)**

Reach 2 33 21.7 <0.0001 DS vs. US 11.1 (-11.5, 33.8)

Year 1 33 294.4 <0.0001

Reach*Year 2 33 5.9  **0.0066**

*Summer Degree Days - Tectah*

Intercept 1 33 78900.6 <0.0001 TH vs. US **77.7 (48.6, 106.8)**

Reach 2 33 0.3 0.7289 DS vs. US **48.1 (12.5, 83.7)**

Year 1 33 57.5 <0.0001

Reach*Year 2 33 15.1 **<0.0001**

*Spring Degree Days – Lost Man*

Intercept 1 5 14830.5 <0.0001 TH vs. US 6.8 (-8.2, 21.8)

Reach 2 5 2.0 0.2303 DS vs. US -2.3 (-17.3, 12.7)

Year 1 5 80.9 0.0003

Reach*Year 2 5 1.3 0.3490

*Summer Degree Days - Lost Man*

Intercept 1 5 20627.2 <0.0001 TH vs. US 11.9 (-5.5, 29.4)

Reach 2 5 2.1 0.2177 DS vs. US 0.4 (-17.0, 17.8)

Year 1 5 44.8 0.0011

Reach*Year 2 5 2.0 0.2315

**S1 Table continued.**

**BACI model BACI differences – Estimate (Lower 95% CI, Upper 95% CI)**

**Response Variable num DF den DF F-statistic p-value Post-hoc test Post-treatment (2017) Post-treatment (2018)**

*Fall Daily Range - Tectah*

Intercept 1 33 313.7 <0.0001 TH vs. US **0.4 (0.2, 0.5)**

Reach 2 33 0.9 0.4023 DS vs. US 0.2 (0.0, 0.3)

Year 1 33 6.9 0.0131

Reach*Year 2 33 11.7 **0.0001**

*Winter Daily Range - Tectah*

Intercept 1 33 476.2 <0.0001 TH vs. US 0.0 (-0.1, 0.1)

Reach 2 33 4.7 0.0162 DS vs. US -0.1 (-0.2, 0.1)

Year 1 33 62.0 <0.0001

Reach*Year 2 33 1.1 0.3339

*Spring Daily Range - Tectah*

Intercept 1 33 262.2 <0.0001 TH vs. US **0.5 (0.2, 0.9)**

Reach 2 33 0.1 0.8632 DS vs. US 0.3 (-0.2, 0.7)

Year 1 33 60.6 <0.0001

Reach*Year 2 33 4.6  **0.0169**

*Summer Daily Range - Tectah*

Intercept 1 33 74.9 <0.0001 TH vs. US **2.5 (1.6, 3.4)**

Reach 2 33 0.0 0.9700 DS vs. US **0.7 (0.0, 1.5)**

Year 1 33 17.2 0.0002

Reach*Year 2 33 16.8 **<0.0001**

*Spring Daily Range – Lost Man*

Intercept 1 5 165.4 0.0001 TH vs. US 0.2 (-0.4, 0.9)

Reach 2 5 0.4 0.6860 DS vs. US -0.1 (-0.6, 0.6)

Year 1 5 1.7 0.2538

Reach*Year 2 5 0.6 0.6069

*Summer Daily Range - Lost Man*

Intercept 1 5 1088.2 <0.0001 TH vs. US 0.1 (-0.2, 0.4)

Reach 2 5 0.2 0.8098 DS vs. US 0.1 (-0.2, 0.4)

Year 1 5 0.3 0.6197

Reach*Year 2 5 0.4 0.6765

**S1 Table continued.**

**BACI model BACI differences – Estimate (Lower 95% CI, Upper 95% CI)**

**Response Variable num DF den DF F-statistic p-value Post-hoc test Post-treatment (2017) Post-treatment (2018)**

*Fall Variance - Tectah*

Intercept 1 33 66.7 <0.0001 TH vs. US **0.1 (0.0, 0.2)**

Reach 2 33 0.3 0.7456 DS vs. US 0.0 (0.0, 0.1)

Year 1 33 2.2 0.1517

Reach*Year 2 33 8.8 **0.0009**

*Winter Variance - Tectah*

Intercept 1 33 129.6 <0.0001 TH vs. US 0.0 (-0.1, 0.1)

Reach 2 33 4.7 0.0158 DS vs. US -0.1 (-0.1, 0.1)

Year 1 33 48.8 <0.0001

Reach*Year 2 33 1.0 0.3947

*Spring Variance - Tectah*

Intercept 1 33 75.2 <0.0001 TH vs. US **0.3 (0.1, 0.5)**

Reach 2 33 0.2 0.8279 DS vs. US 0.2 (-0.1, 0.4)

Year 1 33 29.2 <0.0001

Reach*Year 2 33 4.3 **0.0220**

*Summer Variance - Tectah*

Intercept 1 33 22.7 <0.0001 TH vs. US **1.6 (0.7, 2.5)**

Reach 2 33 0.0 0.9913 DS vs. US **0.5 (0.0, 1.0)**

Year 1 33 4.0 0.0546

Reach*Year 2 33 8.4  **0.0012**

*Spring Variance – Lost Man*

Intercept 1 5 31.0 0.0026 TH vs. US 0.0 (-0.1, 0.1)

Reach 2 5 3.3 0.1197 DS vs. US 0.0 (-0.1, 0.1)

Year 1 5 1.8 0.2363

Reach*Year 2 5 0.0 0.9883

*Summer Variance - Lost Man*

Intercept 1 5 64.0 0.0005 TH vs. US 0.0 (-0.1, 0.1)

Reach 2 5 0.9 0.4777 DS vs. US 0.0 (-0.1, 0.1)

Year 1 5 1.3 0.3119

Reach*Year 2 5 2.0 0.2352
