# Supplementary material for: Shade, light, and stream temperature responses to riparian thinning in second-growth redwood forests of northern California
Source: PLoS One. 2021 Feb 16;16(2):e0246822. doi: 10.1371/journal.pone.0246822 (PMC7886199; doi:10.1371/journal.pone.0246822)
Supplement: S2 Table — (DOCX) [file pone.0246822.s003.docx]

**S2 Table**. **Model selection AIC_c_ table ranking all *a priori* candidate models.**

To better understand the variation in stream thermal regimes and their responses to riparian thinning we considered a range of models in a model selection analysis. In addition to the BACI model of Reach*Year we considered a broad range of environmental covariates that we hypothesized could influence stream temperatures based on previous stream temperature studies. Here we list the covariates we considered in the correlation matrix and whether or not covariates were included in candidate models in model selection. Covariates included in model selection are highlighted in bold and abbreviations of the covariate are listed next to name.

**Effective Shade (ES)** – shade over the stream channel (%). Accounts for the intensity of thinning treatments. Continuous estimates of shade may better represent conditions than categorical fixed effects in BACI model of Reach*Year.

Canopy Closure – another measure of shade over the stream channel (%). Accounts for the intensity of thinning treatments. Did not include in model selection because correlated with Effective Shade.

Light – below canopy light over the stream channel (%). Accounts for the intensity of thinning treatments. Did not include in model selection because correlated with Effective Shade.

**Upstream Temperature (UT)** – summer MWMT from the incoming upstream reach (°C). Upstream thermal conditions could be a major source of non-independent thermal behavior between sites.

**Treatment Proximity (TP)** – categorical variable of whether or not reach is positioned downstream from a thinning treatment (within 400m). Accounts for a lack of independence of some sites that are spatially adjacent.

Air Temperature – summer mean air temperature at the upstream reach within a site (°C). Did not include in model selection because correlated with other physical site characteristics.

**Reach Length (RL)** – length of stream reach (m). Accounts for the fact that the length of the reach may influence the spatial exposure of a reach to thinning.

**Bankfull Width (BW)** – bankfull width of the stream channel (m). Accounts for the fact that the width of the reach may influence the spatial exposure of a reach to thinning.

**Gradient (GR)** – gradient of the stream channel (%). Accounts for the gradient of the stream channel may influence how quickly the water may move through a reach and in turn how quickly it may heat up.

Distance Upstream – position of the reach within the watershed (m). Accounts for the position in the watershed may influence spatial exposure of a reach to thinning. Did not include in model selection because correlated with other physical site characteristics.

Aspect – orientation of the stream channel (°). Accounts for the position in the watershed may influence spatial exposure of a reach to thinning. Did not include in model selection because correlated with other physical site characteristics.

Elevation – elevation of the stream channel (m). Accounts for the position in the watershed may influence spatial exposure of a reach to thinning. Did not include in model selection because correlated with other physical site characteristics.

**Summer MWMT – *Tectah***

**Rank Model *K* df AIC*_c_* ΔAIC*_c_* *w_i_***

1 ES + UT 3 10 104.2 0.0 0.383

2 UT 2 9 106.8 2.6 0.103

3 ES + UT + GR 4 11 107.1 2.9 0.092

4 ES + UT + TP 4 11 107.6 3.4 0.071

5 ES + UT + RL 4 11 107.7 3.4 0.068

6 ES + UT + BW 4 11 107.7 3.5 0.068

7 UT + RL 3 10 108.7 4.5 0.040

8 UT + BW 3 10 109.7 5.5 0.024

9 UT + GR 3 10 109.8 5.6 0.024

10 UT + TP 3 10 109.9 5.7 0.023

11 ES + UT + TP + GR 5 12 110.7 6.5 0.015

12 ES + UT + RL + GR 5 12 110.8 6.6 0.015

13 ES + TP 3 10 110.8 6.6 0.014

14 ES + UT + TP + BW 5 12 111.3 7.0 0.011

15 ES + UT + TP + RL 5 12 111.3 7.1 0.011

16 ES + UT + RL + BW 5 12 111.3 7.1 0.011

17 UT + RL + GR 4 11 112.2 8.0 0.007

18 UT + RL + BW 4 11 112.2 8.0 0.007

19 ES + TP + RL 4 11 113.9 9.7 0.003

20 ES + TP + BW 4 11 114.2 10.0 0.003

21 ES + TP + GR 4 11 114.3 10.1 0.003

22 ES + UT + TP + RL + GR 6 13 114.7 10.5 0.002

23 ES + UT + TP + RL + BW 6 13 115.2 11.0 0.002

24 ES + TP + RL + GR 5 12 117.5 13.3 0.001

25 ES + TP + RL + BW 5 12 117.5 13.3 0.001

26 ES 2 9 119.5 15.3 0.000

27 ES + BW 3 10 122.7 18.4 0.000

28 ES + RL 3 10 122.8 18.6 0.000

29 ES + GR 3 10 122.8 18.6 0.000

30 ES + RL + BW 4 11 126.1 21.9 0.000

31 ES + RL + GR 4 11 126.2 22.0 0.000

32 TP + GR 3 10 129.4 25.2 0.000

33 TP 2 9 131.0 26.8 0.000

34 BACI Model: Reach*Year 4 13 131.3 27.1 0.000

35 TP + BW 3 10 131.8 27.6 0.000

36 TP + RL + GR 4 11 132.3 28.1 0.000

37 TP + RL 3 10 133.4 29.2 0.000

38 TP + RL + BW 4 11 136.6 32.4 0.000

39 Intercept 1 8 139.5 35.3 0.000

40 GR 2 9 140.6 36.4 0.000

41 BW 2 9 142.1 37.9 0.000

42 RL 2 9 142.6 38.4 0.000

43 RL + GR 3 10 143.3 39.1 0.000

44 RL + BW 3 10 145.0 40.8 0.000

**Summer MWAT – *Tectah***

**Rank Model *K* df AIC*_c_* ΔAIC*_c_* *w_i_***

1 ES + UT + TP 4 11 29.3 0.0 0.292

2 ES + UT + RL 4 11 30.8 1.5 0.137

3 ES + UT 3 10 31.1 1.7 0.122

4 ES + TP 3 10 32.0 2.6 0.079

5 ES + UT + TP + RL 5 12 32.1 2.8 0.074

6 ES + UT + TP + GR 5 12 32.4 3.1 0.062

7 ES + UT + TP + BW 5 12 33.0 3.7 0.046

8 ES + UT + RL + GR 5 12 33.7 4.4 0.033

9 ES + UT + BW 4 11 34.0 4.6 0.029

10 ES + UT + RL + BW 5 12 34.5 5.2 0.022

11 ES + UT + GR 4 11 34.6 5.2 0.021

12 ES + TP + RL 4 11 34.9 5.6 0.018

13 ES + TP + BW 4 11 35.0 5.7 0.017

14 ES + UT + TP + RL + GR 6 13 35.2 5.9 0.016

15 ES + TP + GR 4 11 35.3 5.9 0.015

16 ES + UT + TP + RL + BW 6 13 36.0 6.6 0.011

17 ES + TP + RL + BW 5 12 37.9 8.5 0.004

18 ES + TP + RL + GR 5 12 38.4 9.1 0.003

19 UT 2 9 45.6 16.3 0.000

20 UT + RL 3 10 46.7 17.3 0.000

21 UT + TP 3 10 47.4 18.1 0.000

22 UT + GR 3 10 48.8 19.4 0.000

23 UT + BW 3 10 48.8 19.5 0.000

24 UT + RL + GR 4 11 49.6 20.3 0.000

25 UT + RL + BW 4 11 50.0 20.7 0.000

26 ES 2 9 55.4 26.0 0.000

27 TP 2 9 58.3 29.0 0.000

28 ES + GR 3 10 58.5 29.1 0.000

29 ES + BW 3 10 58.6 29.3 0.000

30 ES + RL 3 10 58.7 29.3 0.000

31 TP + RL 3 10 61.3 32.0 0.000

32 TP + BW 3 10 61.5 32.1 0.000

33 TP + GR 3 10 61.6 32.2 0.000

34 ES + RL + GR 4 11 61.9 32.6 0.000

35 ES + RL + BW 4 11 62.1 32.7 0.000

36 BACI Model: Reach*Year 4 13 64.1 34.8 0.000

37 TP + RL + BW 4 11 64.7 35.3 0.000

38 TP + RL + GR 4 11 64.8 35.5 0.000

39 Intercept 1 8 73.9 44.6 0.000

40 GR 2 9 77.0 47.7 0.000

41 RL 2 9 77.0 47.7 0.000

42 BW 2 9 77.0 47.7 0.000

43 RL + GR 3 10 80.3 51.0 0.000

44 RL + BW 3 10 80.3 51.0 0.000

**Summer Degree Days - *Tectah***

**Rank Model *K* df AIC*_c_* ΔAIC*_c_* *w_i_***

1 ES + UT + TP 4 11 406.9 0.0 0.249

2 ES + UT + GR 4 11 406.9 0.0 0.248

3 ES + UT + TP + GR 5 12 408.6 1.6 0.109

4 ES + UT 3 10 409.0 2.1 0.089

5 ES + TP 3 10 409.8 2.9 0.058

6 ES + UT + TP + RL 5 12 410.4 3.5 0.044

7 ES + UT + TP + GR 5 12 410.4 3.5 0.043

8 ES + UT + RL + GR 5 12 410.6 3.7 0.039

9 ES + UT + BW 4 11 411.0 4.1 0.032

10 ES + UT + RL 4 11 412.1 5.2 0.018

11 ES + UT + TP +RL + GR 6 13 412.3 5.4 0.017

12 ES + TP + RL 4 11 412.6 5.7 0.014

13 ES + TP + BW 4 11 412.9 6.0 0.013

14 ES + TP + GR 4 11 413.3 6.4 0.010

15 ES + UT + TP + RL + BW 6 13 414.2 7.3 0.007

16 ES + UT + RL + BW 5 12 414.5 7.6 0.006

17 ES + TP + RL + BW 5 12 416.0 9.1 0.003

18 ES + TP + RL + GR 5 12 416.3 9.4 0.002

19 UT 2 9 424.6 17.7 0.000

20 UT + TP 3 10 425.1 18.2 0.000

21 ES 2 9 425.1 18.2 0.000

22 UT + GR 3 10 425.6 18.7 0.000

23 UT + RL 3 10 427.6 20.7 0.000

24 UT + BW 3 10 427.9 20.9 0.000

25 ES + GR 3 10 427.9 20.9 0.000

26 ES + BW 3 10 428.4 21.5 0.000

27 ES + RL 3 10 428.4 21.5 0.000

28 UT + RL + GR 4 11 429.0 22.0 0.000

29 UT + RL + BW 4 11 431.0 24.1 0.000

30 ES + RL + GR 4 11 431.4 24.4 0.000

31 ES + RL + BW 4 11 431.9 24.9 0.000

32 BACI Model: Reach*Year 4 13 433.4 26.5 0.000

33 TP 2 9 434.3 27.4 0.000

34 TP + BW 3 10 436.7 29.8 0.000

35 TP + RL 3 10 437.3 30.4 0.000

36 TP + GR 3 10 437.5 30.6 0.000

37 TP + RL + BW 4 11 440.1 33.1 0.000

38 TP + RL + GR 4 11 440.7 33.8 0.000

39 Intercept 1 8 447.3 40.4 0.000

40 GR 2 9 449.7 42.8 0.000

41 RL 2 9 450.3 43.4 0.000

42 BW 2 9 450.3 43.4 0.000

43 RL + GR 3 10 453.0 46.1 0.000

44 RL + BW 3 10 453.5 46.6 0.000

**Summer Daily Range – *Tectah***

**Rank Model *K* df AIC*_c_* ΔAIC*_c_* *w_i_***

1 ES + UT 3 10 93.0 0.0 0.286

2 ES + UT + TP + GR 5 12 93.6 0.7 0.203

3 ES 2 9 95.0 2.0 0.105

4 ES + TP 3 10 96.4 3.4 0.052

5 ES + UT + TP 4 11 96.4 3.4 0.051

6 ES + UT + RL 4 11 96.4 3.5 0.050

7 ES + UT + BW 4 11 96.4 3.5 0.050

8 ES + UT + GR 4 11 96.4 3.5 0.050

9 ES + BW 3 10 98.2 5.3 0.020

10 ES + GR 3 10 98.2 5.3 0.020

11 ES + RL 3 10 98.3 5.3 0.020

12 ES + UT + RL + GR 5 12 99.5 6.5 0.011

13 ES + TP + GR 4 11 99.5 6.5 0.011

14 ES + TP + RL 4 11 99.7 6.8 0.009

15 ES + TP + BW 4 11 99.9 6.9 0.009

16 ES + UT + TP + RL 5 12 100.0 7.1 0.008

17 ES + UT + TP + BW 5 12 100.1 7.1 0.008

18 ES + UT + RL + BW 5 12 100.1 7.2 0.008

19 UT 2 9 100.3 7.4 0.007

20 ES + RL + GR 4 11 101.7 8.8 0.003

21 ES + RL + BW 4 11 101.7 8.8 0.003

22 UT + GR 3 10 102.5 9.6 0.002

23 UT + RL 3 10 103.1 10.1 0.002

24 ES + TP + RL + GR 5 12 103.1 10.2 0.002

25 ES + TP + RL + BW 5 12 103.4 10.5 0.002

26 UT + BW 3 10 103.6 10.6 0.001

27 UT + TP 3 10 103.6 10.6 0.001

28 ES + UT + TP + RL + BW 6 13 103.9 11.0 0.001

29 UT + RL + GR 4 11 105.9 12.9 0.000

30 UT + RL + BW 4 11 106.5 13.6 0.000

31 BACI Model: Reach*Year 4 13 108.7 15.7 0.000

32 TP 2 9 109.2 16.2 0.000

33 TP + GR 3 10 111.7 18.7 0.000

34 Intercept 1 8 112.2 19.2 0.000

35 TP + BW 3 10 112.2 19.3 0.000

36 TP + RL 3 10 112.4 19.4 0.000

37 GR 2 9 113.8 20.9 0.000

38 RL 2 9 115.1 22.1 0.000

39 TP + RL + GR 4 11 115.1 22.1 0.000

40 BW 2 9 115.2 22.2 0.000

41 RL + GR 3 10 115.5 22.5 0.000

42 TP + RL + BW 4 11 115.7 22.8 0.000

43 RL + BW 3 10 116.6 23.6 0.000

**Summer Variance – *Tectah***

**Rank Model *K* df AIC*_c_* ΔAIC*_c_* *w_i_***

1 ES + UT + TP + GR 5 12 26.2 0.0 0.873

2 UT 2 9 32.5 6.3 0.038

3 UT + RL + GR 4 11 34.2 7.9 0.016

4 UT + GR 3 10 34.8 8.6 0.012

5 UT + TP 3 10 34.9 8.7 0.011

6 ES + UT 3 10 35.2 9.0 0.010

7 ES + UT + RL + GR 5 12 35.3 9.1 0.009

8 UT + BW 3 10 35.8 9.6 0.007

9 UT + RL 3 10 35.8 9.6 0.007

10 ES 2 9 37.8 11.6 0.003

11 ES + UT + GR 4 11 37.9 11.7 0.003

12 ES + UT + TP 4 11 38.3 12.1 0.002

13 ES + UT + RL 4 11 38.7 12.4 0.002

14 ES + UT + BW 4 11 38.7 12.5 0.002

15 UT + RL + BW 4 11 39.3 13.0 0.001

16 ES + RL 3 10 40.6 14.3 0.001

17 ES + GR 3 10 40.9 14.7 0.001

18 ES + TP 3 10 41.0 14.8 0.001

19 ES + BW 3 10 41.1 14.9 0.001

20 ES + UT + TP + RL 5 12 41.6 15.4 0.000

21 ES + UT + TP + BW 5 12 41.9 15.7 0.000

22 ES + UT + RL + BW 5 12 42.3 16.1 0.000

23 TP 2 9 43.6 17.4 0.000

24 ES + TP + RL 4 11 43.7 17.5 0.000

25 ES + RL + GR 4 11 44.0 17.8 0.000

26 ES + RL + BW 4 11 44.0 17.8 0.000

27 ES + TP + GR 4 11 44.2 18.0 0.000

28 Intercept 1 8 44.2 18.0 0.000

29 ES + TP + BW 4 11 44.5 18.2 0.000

30 GR 2 9 45.2 18.9 0.000

31 TP + GR 3 10 45.2 19.0 0.000

32 ES + UT + TP + RL + BW 6 13 45.5 19.3 0.000

33 TP + BW 3 10 46.4 20.2 0.000

34 RL + GR 3 10 46.7 20.5 0.000

35 BW 2 9 47.1 20.9 0.000

36 RL 2 9 47.1 20.9 0.000

37 BACI Model: Reach*Year 4 13 47.3 21.1 0.000

38 ES + TP + RL + GR 5 12 47.4 21.2 0.000

39 ES + TP + RL + BW 5 12 47.4 21.2 0.000

40 TP + RL 3 10 47.5 21.3 0.000

41 RL + BW 3 10 48.4 22.1 0.000

42 TP + RL + GR 4 11 48.5 22.3 0.000

43 TP + RL + BW 4 11 50.7 24.5 0.000

**Summer MWMT – *Lost Man***

**Rank Model *K* df AIC*_c_* ΔAIC*_c_* *w_i_***

1 Intercept 1 3 -6.1 0.0 0.256

2 GR 2 4 -5.4 0.7 0.178

3 UT 2 4 -4.5 1.6 0.112

4 BW 2 4 -4.4 1.7 0.109

5 UT + GR 3 5 -4.2 1.9 0.097

6 UT + BW 3 5 -3.0 3.1 0.054

7 ES 2 4 -2.5 3.6 0.042

8 TP 2 4 -1.4 4.7 0.025

9 RL 2 4 -1.4 4.7 0.024

10 TP + GR 3 5 -1.4 4.8 0.024

11 TP + BW 3 5 -0.8 5.3 0.018

12 RL + BW 3 5 -0.1 6.1 0.012

13 RL + GR 3 5 0.8 7.0 0.008

14 ES + GR 3 5 0.9 7.0 0.008

15 ES + BW 3 5 1.4 7.6 0.006

16 ES + UT 3 5 1.7 7.9 0.005

17 UT + TP 3 5 1.8 7.9 0.005

18 UT + RL 3 5 1.8 7.9 0.005

19 ES + RL 3 5 3.4 9.6 0.002

20 ES + TP 3 5 3.7 9.9 0.002

21 ES + UT + GR 4 6 4.1 10.2 0.002

22 UT + RL + BW 4 6 4.3 10.4 0.001

23 TP + RL + BW 4 6 4.5 10.7 0.001

24 UT + RL + GR 4 6 4.6 10.7 0.001

25 TP + RL 3 5 4.8 11.0 0.001

26 ES + UT + BW 4 6 5.8 11.9 0.001

27 ES + TP + BW 4 6 7.2 13.4 0.000

28 TP + RL + GR 4 6 7.4 13.5 0.000

29 ES + TP + GR 4 6 7.4 13.6 0.000

30 ES + RL + BW 4 6 8.7 14.9 0.000

31 ES + RL + GR 4 6 9.6 15.8 0.000

32 ES + UT + TP 4 6 10.5 16.6 0.000

33 ES + UT + RL 4 6 10.5 16.6 0.000

34 ES + TP + RL 4 6 12.2 18.3 0.000

35 ES + UT + TP + GR 5 7 15.2 21.4 0.000

36 ES + UT + TP + BW 5 7 16.6 22.7 0.000

37 ES + UT + RL + BW 5 7 16.9 23.1 0.000

38 ES + UT + RL + GR 5 7 17.3 23.4 0.000

39 ES + TP + RL + BW 5 7 17.7 23.9 0.000

40 ES + TP + RL + GR 5 7 20.6 26.7 0.000

41 ES + UT + TP + RL 5 7 23.7 29.8 0.000

42 BACI Model: Reach*Year 4 8 33.6 39.7 0.000

43 ES + UT + TP + RL + BW 6 8 35.2 41.3 0.000

44 ES + UT + TP + RL + GR 6 8 37.2 43.3 0.000

**Summer MWAT – *Lost Man***

**Rank Model *K* df AIC*_c_* ΔAIC*_c_* *w_i_***

1 GR 2 4 -13.4 0.0 0.228

2 Intercept 1 3 -13.0 0.3 0.193

3 BW 2 4 -12.7 0.7 0.161

4 UT 2 4 -11.2 2.2 0.077

5 TP + GR 3 5 -10.7 2.6 0.061

6 TP + BW 3 5 -10.4 2.9 0.052

7 ES 2 4 -10.2 3.1 0.047

8 UT + BW 3 5 -9.6 3.8 0.034

9 TP 2 4 -8.9 4.4 0.025

10 UT + BW 3 5 -8.9 4.5 0.024

11 RL 2 4 -8.3 5.0 0.019

12 ES + BW 3 5 -8.0 5.4 0.016

13 RL + BW 3 5 -7.6 5.7 0.013

14 ES + GR 3 5 -7.6 5.7 0.013

15 RL + GR 3 5 -7.1 6.3 0.010

16 UT + TP 3 5 -5.5 7.8 0.005

17 ES + UT 3 5 -5.1 8.3 0.004

18 UT + RL 3 5 -4.9 8.4 0.003

19 ES + TP 3 5 -4.7 8.6 0.003

20 ES + TP + BW 4 6 -4.5 8.9 0.003

21 TP + RL + BW 4 6 -4.4 8.9 0.003

22 ES + RL 3 5 -4.4 9.0 0.003

23 ES + TP + GR 4 6 -2.7 10.6 0.001

24 TP + RL 3 5 -2.7 10.7 0.001

25 TP + RL + GR 4 6 -1.9 11.4 0.001

26 UT + RL + BW 4 6 -1.0 12.4 0.001

27 ES + UT + GR 4 6 -0.9 12.4 0.001

28 ES + UT + BW 4 6 -0.8 12.6 0.000

29 UT + RL + GR 4 6 -0.8 12.6 0.000

30 ES + RL + BW 4 6 0.4 13.8 0.000

31 ES + RL + GR 4 6 0.9 14.3 0.000

32 ES + UT + TP 4 6 3.0 16.3 0.000

33 ES + TP + RL 4 6 3.6 17.0 0.000

34 ES + UT + RL 4 6 3.7 17.0 0.000

35 ES + TP + RL + BW 5 7 7.5 20.9 0.000

36 ES + UT + TP + BW 5 7 7.7 21.1 0.000

37 ES + UT + TP + GR 5 7 8.7 22.0 0.000

38 ES + TP + RL + GR 5 7 10.2 23.6 0.000

39 ES + UT + RL + BW 5 7 12.0 25.4 0.000

40 ES + UT + RL + GR 5 7 12.2 25.6 0.000

41 ES + UT + TP + RL 5 7 16.1 29.5 0.000

42 BACI Model: Reach*Year 4 8 22.3 35.6 0.000

40 ES + UT + TP + RL + BW 6 8 28.6 42.0 0.000

44 ES + UT + TP + RL + GR 6 8 30.6 44.0 0.000

**Summer Degree Days – *Lost Man***

**Rank Model *K* df AIC*_c_* ΔAIC*_c_* *w_i_***

1 UT 2 4 103.5 0.0 0.195

2 Intercept 1 3 103.8 0.3 0.169

3 ES 2 4 104.3 0.8 0.134

4 GR 2 4 104.6 1.1 0.114

5 BW 2 4 105.2 1.7 0.083

6 TP + GR 3 5 106.9 3.4 0.036

7 ES + BW 3 5 107.3 3.8 0.030

8 TP 2 4 107.3 3.8 0.029

9 TP + BW 3 5 107.4 3.9 0.028

10 UT + GR 3 5 108.1 4.6 0.020

11 ES + GR 3 5 108.1 4.6 0.020

12 UT + TP 3 5 108.2 4.7 0.019

13 RL 2 4 108.4 4.9 0.017

14 ES + TP 3 5 108.4 4.9 0.017

15 ES + TP + BW 4 6 108.4 4.9 0.017

16 UT + BW 3 5 108.6 5.0 0.016

17 ES + UT 3 5 108.6 5.1 0.016

18 UT + RL 3 5 109.6 6.1 0.009

19 ES + RL 3 5 109.8 6.2 0.009

20 RL + BW 3 5 110.4 6.9 0.006

21 RL + GR 3 5 110.8 7.3 0.005

22 ES + TP + GR 4 6 111.4 7.9 0.004

23 TP + RL 3 5 113.4 9.9 0.001

24 TP + RL + BW 4 6 113.7 10.2 0.001

25 ES + UT + BW 4 6 114.5 11.0 0.001

26 ES + UT + GR 4 6 114.9 11.3 0.001

27 ES + UT + TP 4 6 115.5 12.0 0.001

28 TP + RL + GR 4 6 115.6 12.1 0.001

29 ES + RL + BW 4 6 116.1 12.5 0.000

30 ES + TP + RL 4 6 116.3 12.7 0.000

31 ES + RL + GR 4 6 116.4 12.9 0.000

32 UT + RL + BW 4 6 116.7 13.1 0.000

33 UT + RL + GR 4 6 116.8 13.2 0.000

34 ES + UT + RL 4 6 117.4 13.8 0.000

35 ES + UT + TP + BW 5 7 120.7 17.2 0.000

36 ES + TP + RL + BW 5 7 121.3 17.8 0.000

37 ES + UT + TP + GR 5 7 122.8 19.3 0.000

38 ES + TP + RL + GR 5 7 124.0 20.5 0.000

39 ES + UT + RL + BW 5 7 127.7 24.2 0.000

40 ES + UT + RL + GR 5 7 127.8 24.3 0.000

41 ES + UT + TP + RL 5 7 128.7 25.1 0.000

42 BACI Model: Reach*Year 4 8 134.3 30.8 0.000

43 ES + UT + TP + RL + BW 6 8 142.4 38.9 0.000

44 ES + UT + TP + RL + GR 6 8 144.5 40.9 0.000

**Summer Daily Range – *Lost Man***

**Rank Model *K* df AIC*_c_* ΔAIC*_c_* *w_i_***

1 Intercept 1 3 -24.5 0.0 0.543

2 ES 2 4 -20.8 3.8 0.083

3 TP 2 4 -20.6 4.0 0.075

4 RL 2 4 -20.4 4.1 0.069

5 BW 2 4 -20.4 4.1 0.068

6 UT 2 4 -20.0 4.6 0.055

7 GR 2 4 -19.9 4.6 0.054

8 ES + TP 3 5 -15.3 9.2 0.005

9 UT + BW 3 5 -15.0 9.5 0.005

10 TP + RL 3 5 -15.0 9.6 0.005

11 ES + BW 3 5 -14.9 9.6 0.004

12 ES + RL 3 5 -14.6 9.9 0.004

13 ES + GR 3 5 -14.6 9.9 0.004

14 TP + BW 3 5 -14.6 10.0 0.004

15 ES + UT 3 5 -14.5 10.0 0.004

16 RL + BW 3 5 -14.5 10.1 0.004

17 UT + TP 3 5 -14.4 10.1 0.004

18 TP + GR 3 5 -14.3 10.2 0.003

19 RL + GR 3 5 -14.3 10.3 0.003

20 UT + RL 3 5 -14.3 10.3 0.003

21 UT + GR 3 5 -14.0 10.5 0.003

22 ES + TP + BW 4 6 -6.7 17.8 0.000

23 ES + TP + RL 4 6 -6.7 17.8 0.000

24 ES + UT + BW 4 6 -6.6 17.9 0.000

25 ES + UT + TP 4 6 -6.6 18.0 0.000

26 ES + TP + GR 4 6 -6.5 18.0 0.000

27 UT + RL + BW 4 6 -6.4 18.1 0.000

28 TP + RL + BW 4 6 -6.3 18.2 0.000

29 TP + RL + GR 4 6 -6.2 18.3 0.000

30 ES + RL + BW 4 6 -6.2 18.4 0.000

31 ES + UT + GR 4 6 -6.0 18.6 0.000

32 ES + RL + GR 4 6 -5.9 18.6 0.000

33 UT + RL + GR 4 6 -5.9 18.6 0.000

34 ES + UT + RL 4 6 -5.9 18.7 0.000

35 ES + UT + TP + BW 5 7 6.2 30.7 0.000

36 ES + TP + RL + BW 5 7 6.4 30.9 0.000

37 ES + UT + TP + RL 5 7 6.5 31.0 0.000

38 ES + TP + RL + GR 5 7 6.5 31.0 0.000

39 ES + UT + RL + BW 5 7 6.5 31.1 0.000

40 ES + UT + TP + GR 5 7 6.6 31.6 0.000

41 ES + UT + RL + GR 5 7 7.0 31.6 0.000

42 BACI Model: Reach*Year 4 8 27.7 52.2 0.000

43 ES + UT + TP + RL + BW 6 8 28.1 52.6 0.000

44 ES + UT + TP + RL + GR 6 8 28.4 52.9 0.000

**Summer Variance – *Lost Man***

**Rank Model *K* df AIC*_c_* ΔAIC*_c_* *w_i_***

1 Intercept 1 3 -72.6 0.0 0.221

2 BW 2 4 -72.0 0.6 0.163

3 ES 2 4 -71.2 1.4 0.108

4 ES + GR 3 5 -70.4 2.2 0.073

5 GR 2 4 -70.1 2.5 0.064

6 TP 2 4 -69.7 2.9 0.052

7 ES + BW 3 5 -69.7 2.9 0.051

8 ES + UT 3 5 -69.5 3.1 0.047

9 RL 2 4 -69.5 3.1 0.047

10 ES + UT + BW 4 6 -69.3 3.3 0.042

11 UT 2 4 -68.5 4.1 0.029

12 ES + TP 3 5 -67.6 5.0 0.018

13 ES + UT + BW 4 6 -67.1 5.5 0.014

14 UT + BW 3 5 -67.0 5.6 0.014

15 RL + BW 3 5 -65.7 6.9 0.007

16 TP + BW 3 5 -65.7 6.9 0.007

17 TP + RL 3 5 -65.6 7.0 0.007

18 RL + GR 3 5 -65.6 7.0 0.007

19 UT + GR 3 5 -65.3 7.2 0.006

20 ES + RL 3 5 -65.1 7.5 0.005

21 ES + UT + TP 4 6 -64.0 8.6 0.003

22 UT + TP 3 5 -64.0 8.6 0.003

23 UT + RL 3 5 -63.8 8.8 0.003

24 TP + GR 3 5 -63.6 9.0 0.003

25 ES + RL + BW 4 6 -62.1 10.5 0.001

26 ES + TP + GR 4 6 -61.7 10.8 0.001

27 ES + RL + GR 4 6 -61.7 10.9 0.001

28 ES + UT + RL 4 6 -61.0 11.6 0.001

29 ES + TP + BW 4 6 -60.9 11.7 0.001

30 ES + TP + RL 4 6 -59.2 13.4 0.000

31 UT + RL + GR 4 6 -58.4 14.2 0.000

32 UT + RL + BW 4 6 -58.3 14.3 0.000

33 ES + UT + TP + GR 5 7 -57.4 15.2 0.000

34 TP + RL + GR 4 6 -57.1 15.5 0.000

35 TP + RL + BW 4 6 -56.8 15.8 0.000

36 ES + UT + RL + GR 5 7 -56.1 16.5 0.000

37 ES + UT + RL + BW 5 7 -55.9 16.7 0.000

38 ES + UT + TP + BW 5 7 -54.8 17.8 0.000

39 ES + UT + TP + RL 5 7 -50.8 21.8 0.000

40 ES + TP + RL + BW 5 7 -48.9 23.7 0.000

41 ES + TP + RL + GR 5 7 -48.6 24.0 0.000

42 ES + UT + TP + RL + GR 6 8 -35.5 37.1 0.000

43 ES + UT + TP + RL + GR 6 8 -34.6 38.0 0.000

44 BACI Model: Reach*Year 4 8 -26.3 46.3 0.000
