# Supplementary material for: Shade, light, and stream temperature responses to riparian thinning in second-growth redwood forests of northern California
Source: PLoS One. 2021 Feb 16;16(2):e0246822. doi: 10.1371/journal.pone.0246822 (PMC7886199; doi:10.1371/journal.pone.0246822)
Supplement: S3 Table — (DOCX) [file pone.0246822.s004.docx]

**S3 Table**. **Best supported models determined by model selection.** Best supported linear mixed-effects models where fixed and random effects are determined by model selection for each response variable.

**Summer MWMT - Tectah**

Summer MWMT ~ Effective Shade + UpstreamTemp with Random Effect of Site fit by Restricted Maximum Likelihood that includes weights argument that allows Reach and Year to vary.

Fixed Effects Random Effects

Value SE DF t-value p-value Groups SD

Intercept 18.86 1.89 36 10.01 <0.0001 Site 0.317

EffectiveShade -0.10 0.01 36 -7.92 <0.0001 Residual 0.509

UpstreamTemp 0.43 0.08 36 5.66 <0.0001

**Summer MWAT - Tectah**

Summer MWAT ~ Effective Shade + UpstreamTemp + TreatmentProximity with Random Effect of Site fit by Restricted Maximum Likelihood that includes weights argument that allows Reach and Year to vary.

Fixed Effects Random Effects

Value SE DF t-value p-value Groups SD

Intercept 15.31 0.73 35 21.10 <0.0001 Site 0.018

EffectiveShade -0.03 0.00 35 -13.42 <0.0001 Residual 0.228

UpstreamTemp 0.13 0.05 35 2.65 0.0119

TreatmentProximity 0.43 0.10 35 4.42 0.0001

**Summer Degree Days - Tectah**

Summer Degree Days ~ Effective Shade + UpstreamTemp + TreatmentProximity with Random Effect of Site fit by Restricted Maximum Likelihood that includes weights argument that allows Reach and Year to vary.

Fixed Effects Random Effects

Value SE DF t-value p-value Groups SD

Intercept 1329.03 48.79 35 27.24 <0.0001 Site 5.493

EffectiveShade -3.22 0.27 35 -11.73 <0.0001 Residual 18.755

UpstreamTemp 8.21 2.84 35 2.89 0.0066

TreatmentProximity 17.40 7.01 35 2.48 0.0180

**Summer Daily Range - Tectah**

Summer Daily Range ~ Effective Shade + UpstreamTemp with Random Effect of Site fit by Restricted Maximum Likelihood that includes weights argument that allows Reach and Year to vary.

Fixed Effects Random Effects

Value SE DF t-value p-value Groups SD

Intercept 8.26 1.77 36 4.77 <0.0001 Site 0.357

EffectiveShade -0.10 0.01 36 -7.55 <0.0001 Residual 0.390

UpstreamTemp 0.17 0.07 36 2.58 0.0142

**Summer Variance - Tectah**

Summer Variance ~ EffectiveShade + UpstreamTemp + TreatmentProximity + Gradient with Random Effect of Site fit by Restricted Maximum Likelihood that includes weights argument that allows Reach and Year to vary.

Fixed Effects Random Effects

Value SE DF t-value p-value Groups SD

Intercept -0.07 0.42 34 -0.16 0.8771 Site 0.239

EffectiveShade -0.02 0.00 34 -4.61 0.0001 Residual 0.181

UpstreamTemp 0.13 0.01 34 11.73 <0.0001

TreatmentProximity -0.14 0.02 34 -6.65 <0.0001

Gradient 0.05 0.01 34 7.91 <0.0001

**Summer MWMT – Lost Man**

Summer MWMT ~ Intercept with Random Effect of Site fit by Restricted Maximum Likelihood.

Fixed Effects Random Effects

Value SE DF t-value p-value Groups SD

Intercept 13.58 0.16 10 83.41 <0.0001 Intercept 0.226

Residual 0.103

**Summer MWAT – Lost Man**

Summer MWMT ~ Gradient with Random Effect of Site fit by Restricted Maximum Likelihood.

Fixed Effects Random Effects

Value SE DF t-value p-value Groups SD

Intercept 12.56 0.16 9 78.52 <0.0001 Intercept 0.005

Gradient 0.10 0.03 9 3.21 0.0107 Residual 0.086

**Summer Degree Days – Lost Man**

Summer MWMT ~ UpstreamTemp with Random Effect of Site fit by Restricted Maximum Likelihood.

Fixed Effects Random Effects

Value SE DF t-value p-value Groups SD

Intercept 836.74 112.74 9 7.42 <0.0001 Intercept 0.552

UpstreamTemp 21.11 8.29 9 2.55 0.0314 Residual 11.178

**Summer Daily Range – Lost Man**

Summer MWMT ~ Intercept with Random Effect of Site fit by Restricted Maximum Likelihood.

Fixed Effects Random Effects

Value SE DF t-value p-value Groups SD

Intercept 0.72 0.02 10 39.76 <0.0001 Intercept 0.001

Residual 0.062

**Summer Variance – Lost Man**

Summer MWMT ~ Intercept with Random Effect of Site fit by Restricted Maximum Likelihood.

Fixed Effects Random Effects

Value SE DF t-value p-value Groups SD

Intercept 0.07 0.01 10 7.99 <0.0001 Intercept 0.012

Residual 0.007
