# Supplementary material for: Shade, light, and stream temperature responses to riparian thinning in second-growth redwood forests of northern California
Source: PLoS One. 2021 Feb 16;16(2):e0246822. doi: 10.1371/journal.pone.0246822 (PMC7886199; doi:10.1371/journal.pone.0246822)
Supplement: S1 File — (PDF) [file pone.0246822.s005.pdf]

S1 File - Reference sites analysis.

Evaluating the effectiveness of riparian buffer prescriptions relative to reference conditions for riparian shade, light, and stream temperature in small streams in northern California

Introduction

In this analysis we evaluated the effectiveness of experimental riparian buffer protections that follow the prescriptions described by the Aquatic Habitat Conservation Plan (AHCP) negotiated between Green Diamond Resource Company and the National Marine Fisheries Service and United States Fish and Wildlife Service (Green Diamond Resource Company 2006). We compared how these AHCP buffers influenced riparian shade, light, and stream temperature relative to intact forest conditions. AHCP buffer prescriptions included a single-sided 150 foot (~45 m) wide buffer zone that consisted of a 22.5 m wide inner zone of 85% canopy retention and a 22.5 m wide outer zone of 70% canopy retention (Green Diamond Resource Company 2006). Because the buffer treatment was only applied to one side and the other side was intact forest, we hypothesized that the AHCP buffer would result in minimal changes in shade, light, and stream temperature and would not differ relative to intact forest conditions.

Methods

We compared stream reaches lined by AHCP riparian buffer conditions to reaches lined by intact forests. AHCP buffers occurred adjacent to upslope harvest units while intact forest reaches occurred outside of harvest units. Stream reaches were 150-200 m in length.

We collected data following a Before-After-Control-Impact study design where pre-treatment data collection occurred in 2016, harvest occurred in 2017, and post-treatment data collection occurred in 2018. We examined before-after responses in AHCP buffer and intact forest reference reaches located in the West and East Forks of Tectah Creek Watersheds. AHCP buffered reaches and intact forest reference reaches occurred upstream and downstream of experimental thinning reaches inside and outside of harvest units for a total sample size of 16 (Table 1). We pooled upstream and downstream reaches for comparisons of riparian shade and light, but because downstream temperature responses could be differentially influenced by experimental thinning reaches we examined temperature responses in upstream and downstream reaches separately.

We measured riparian shade using hemispherical photography. We considered canopy closure and effective shade to evaluate differences in riparian shade. We measured below-canopy light using pyranometers. We measured stream temperature with digital temperature sensors. We evaluated stream temperature as the summer Maximum Weekly Average of the Maximum (MWMT) as an indication of magnitude. See main paper for more details about data collection methods. We compared before-after differences of riparian shade, light, and stream temperature conditions between AHCP and intact forest reaches using mean estimates, non-parametric bootstrapped 95% confidence intervals computed using the boot package in R (Canty et al. 2020), and non-parametric Kolmogorov-Smirnov tests ($\alpha = 0.05$). All graphics and analyses were conducted in R (R Core Team 2020).

Table 1. Distribution of sample sizes of AHCP buffered reaches and intact forest reference reaches in upstream and downstream reaches.

Buffer Type	Upstream	Downstream	All Reaches
AHCP	5	3	8
Intact Forest	3	5	8
Total	8	8	16

Results

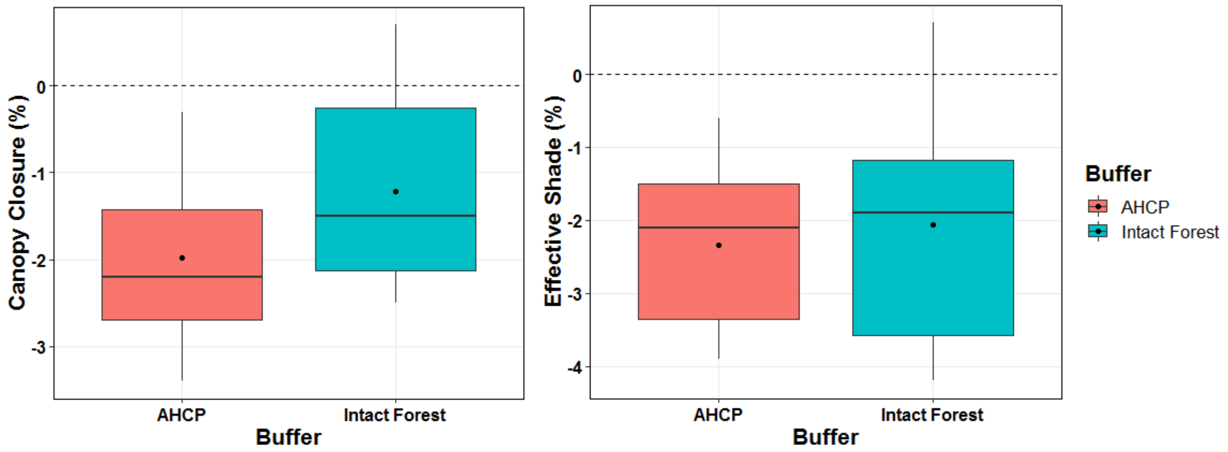

Fig 1. Boxplots of before-after differences in riparian shade in AHCP buffered and intact forest reaches as a) canopy closure (%), and b) effective shade (%). Black dots indicate mean values.

Analyses indicated that riparian shade did not statistically differ between AHCP buffered and intact forest reaches, and that differences overall were small. Canopy closure decreased in AHCP buffered reaches by a mean of 2.0% (95% CI: -2.7, -1.2) and intact forest reaches by a mean of 1.2% (95% CI: -2.0, -0.4) (Fig 1a), but did not differ between buffer types (K-S test: $p = 0.627$). Effective shade decreased in AHCP buffered reaches by a mean of 2.3% (95% CI: -3.1, -1.6) and intact forest reaches by a mean of 2.1% (95% CI: -3.2, -0.9) (Fig 1b), but also did not differ between buffer types (K-S test: $p = 0.964$).

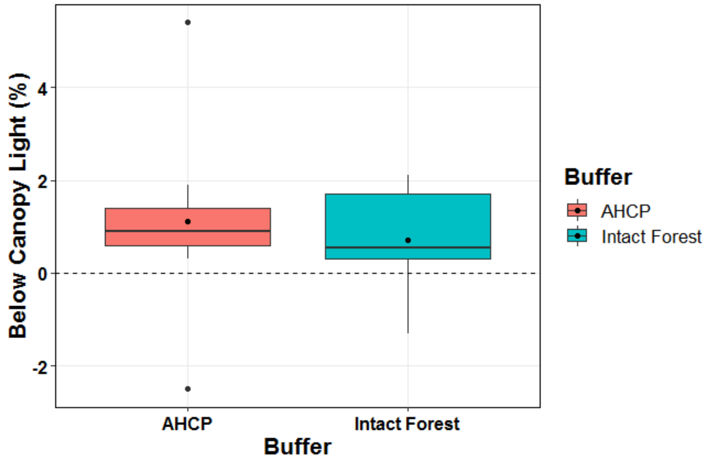

Fig 2. Boxplots of before-after differences in below-canopy light (%) in AHCP buffered and intact forest reaches. Black dots within boxplots indicate mean values and black dots outside boxplots indicate outliers.

Below-canopy light increased in AHCP buffered reaches by a mean of 1.1% (95% CI: -0.4, 2.8) and intact forest reaches by a mean of 0.7% (95% CI: -0.2, 1.6) (Fig 2), but did not differ between buffer types (K-S test: $p = 0.737$).

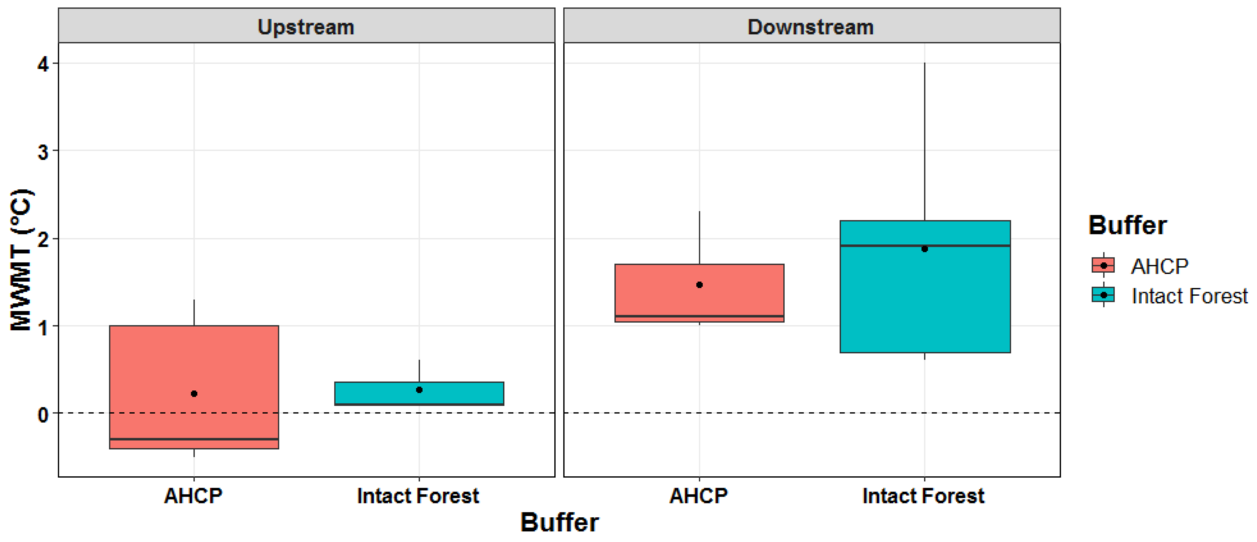

Fig 3. Boxplots of before-after differences in stream temperature as summer MWMT (°C) in AHCP buffered and intact forest reaches in a) upstream sites and b) downstream reaches. Black dots indicate mean values.

Stream temperature responses as summer MWMT increased in both upstream and downstream reaches but did not differ between buffer types (Fig 3). In upstream reaches, stream temperature in AHCP buffered reaches increased by a mean of 0.2°C (95% CI: -0.4, 0.9) and by a mean of 0.3°C (95% CI: 0.1, 0.6) in intact forest reaches (Fig 3a), but did not differ between buffer types (K-S test: $p = 0.464$). In downstream reaches, stream temperature increased in AHCP buffered reaches by a mean of 1.5°C (95% CI: 1.0, 2.3) and by a mean of 1.9°C (95% CI: 0.9, 3.0) in intact forest reaches (Fig 3b) and did not differ between buffer types (K-S test: $p = 0.857$).

Discussion

Recent research has shown that riparian buffers can be effective management strategies for protecting riparian forests and minimizing impacts to stream temperature (Moore et al. 2005). However, stream temperature responses to riparian buffers continue to vary depending on their implementation (Moore et al. 2005, Gomi et al. 2006, Groom et al. 2011, Janisch et al. 2012, Bladon et al. 2016). In this analysis we evaluated the effectiveness of Green Diamond Resource Company's one-sided 45 m AHCP buffer prescriptions relative to intact forest conditions. We observed small reductions in shade, increases in light, and increases in stream temperature, but none of these responses differed between AHCP buffers and intact forest reaches, supporting our hypotheses and predictions.

Reductions in riparian shade were minor and did not differ between buffer types (intact forest vs. AHCP buffer). Measured differences in riparian shade were slightly greater as canopy closure than effective shade in AHCP buffered reaches relative to intact forest reaches. However, both of these differences were minor (all less than 1%) and did not differ significantly between buffer types. Reductions in shade measured in this study were substantially less than what has been documented with historical timber harvest practices (Moore et al. 2005) and were more consistent with what has been documented with recent contemporary buffer studies (Groom et al. 2011, Bladon et al. 2016), especially with wider buffer prescriptions (e.g. >15 m). This is likely due to the width of the buffer prescriptions (~45m) and the fact that the buffer treatment was only applied to one side of the stream channel (the other side was lined by intact forest). The small reductions in shade we observed in both buffer types could be due to edge effects

from adjacent thinning treatments. Alternatively, it could be due to inherent interannual variation such as storm events that caused individual trees to fall within the riparian zone of these forests.

Increases in light reflected the direction and magnitude of shade responses. Although percent light levels increased slightly more in AHCP buffered reaches than intact forest reaches, these differences were very small (<0.5%) and did not differ significantly between reach types. Few stream temperature studies have directly measured solar radiation, but Kiffney et al. (2003) similarly did not observe differences between 30 m buffers and control treatments. Similar to the riparian shade responses, increases in light observed in both reach types could be explained by edge effects of adjacent thinning treatments or interannual variation.

We observed distinct stream temperature responses in the upstream and downstream reaches. Maximum temperatures increased slightly in both reach types in upstream reaches, but the magnitude of these increases were small (<1.0°C), and did not differ between buffer types. Documented temperature responses were comparable to what have been observed with buffers of similar width (Groom et al. 2011, Bladon et al. 2016). The fact that we documented increases in temperature in both buffer types could suggest that 2018 was a warmer year than 2016. Alternatively, because multiple thinning treatments occurred within these watersheds, increases in temperature in upstream reaches could be due to the downstream transport of heat from thinning treatments positioned higher in the watershed (Moore et al. 2005). Although we observed a wider range of values in AHCP buffered reaches than in intact forest reaches, this is more likely due to the fact that more sites were influenced by the downstream transport of heat rather than differences in local buffer conditions.

Temperature responses in downstream reaches were larger than in upstream reaches (95% CI's ranged 1-3°C) but did not differ between buffer types. As a result, increases in temperature in downstream reaches were unlikely due to differences in riparian buffer prescriptions, but more likely to downstream transport of temperature increases observed in intensive experimental thinning reaches (Moore et al. 2005). Downstream temperature responses were slightly higher in intact forest reaches than in AHCP buffered reaches, but this is more likely due to differences in the downstream transport of heat from thinned reaches than due to local differences in riparian forest conditions.

In conclusion, we observed that the AHCP buffer prescriptions resulted in minimal changes in riparian shade, light, and stream temperature and those responses did not differ relative to intact forest conditions. These results suggest that the AHCP buffer prescriptions may offer similar protections to intact second-growth forests for the response variables we considered. These findings support previous research that has documented that riparian buffers can act as effective management strategies for riparian forests and instream conditions.

References

Bladon, K.D., N.A. Cook, J.T. Light, and C. Segura. 2016. A catchment-scale assessment of stream temperature response to contemporary forest harvesting in the Oregon Coast Range. Forest Ecology and Management 379: 153-164.

Canty, A., and B.D. Ripley. 2020. boot: bootstrap functions. R package version 1.3-25 https://CRAN.R-project.org/package=boot

Gomi, T., R.D. Moore, and A.S. Dhakal. 2006. Headwater stream temperature response to clear-cut harvesting with different riparian treatments, coastal British Columbia, Canada. Water Resources Research 42: W08437.

Green Diamond Resource Company. 2006. Aquatic Habitat Conservation Plan and Candidate Conservation Agreement with Assurances. Volume 1, 552 p.

Groom, J.D., L. Dent, L.J. Madsen, and J. Fleuret. 2011. Response of western Oregon (USA) stream temperatures to contemporary forest management. Forest Ecology and Management 262: 1618-1629.

Janisch, J.E., S.M. Wondzell, and W.J. Ehinger. 2012. Headwater stream temperature: Interpreting response after logging with and without riparian buffers, Washington, USA. Forest Ecology and Management 270:302-313.

Kiffney, P.M., J.S. Richardson, and J.P Bull. 2004. Responses of periphyton and insects to experimental manipulation of riparian buffer width along forest streams. Journal of Applied Ecology 40: 1060-1076.

Moore, R.D., D.L. Spittlehouse, and A. Story. 2005. Riparian microclimate and stream temperature response to forest harvesting: a review. Journal of the American Water Resources Association. 41: 813-834.

R Core Team. 2020. R: A language and environment for statistical computing. R Foundation for Statistical Computing, Vienna, Austria.
